# Movement markers of schizophrenia: a detailed analysis of patients’ gait patterns

**DOI:** 10.1007/s00406-022-01402-y

**Published:** 2022-04-01

**Authors:** Lily Martin, Kevin Stein, Katharina Kubera, Nikolaus F. Troje, Thomas Fuchs

**Affiliations:** 1 Department of Psychology, Faculty of Behavioural and Cultural Studies, Heidelberg University, Heidelberg, Germany; 2 Optimization, Robotics and Biomechanics, ZITI-Institute of Computer Engineering, Heidelberg University, Heidelberg, Germany; 3 Department of General Psychiatry, Centre for Psychosocial Medicine, Academic Medical Center, Heidelberg University, Voßstr., 69115 Heidelberg, Germany; 4 BioMotionLab, Department of Biology, Centre for Vision Research, York University, Toronto, Canada

**Keywords:** Motion capture, Motor abnormalities, Movement, Controlled trial, Embodiment

## Abstract

Motor abnormalities occur in the majority of persons with schizophrenia but are generally neglected in clinical care. Psychiatric diagnostics fail to include quantifiable motor variables and few assessment tools examine full-body movement. We assessed full-body movement during gait of 20 patients and 20 controls with motion capture technology, symptom load (PANSS, BPRS) and Neurological Soft Signs (NSS). In a data-driven analysis, participants’ motion patterns were quantified and compared between groups. Resulting movement markers (MM) were correlated with the clinical assessment. We identified 16 quantifiable MM of schizophrenia. While walking, patients and controls display significant differences in movement patterns related to posture, velocity, regularity of gait as well as sway, flexibility and integration of body parts. Specifically, the adjustment of body sides, limbs and movement direction were affected. The MM remain significant when controlling for medication load. They are systematically related to NSS. Results add assessment tools, analysis methods as well as theory-independent MM to the growing body of research on motor abnormalities in schizophrenia.

**Supplementary Information:**

The online version contains supplementary material available at 10.1007/s00406-022-01402-y.

## Introduction

Genuine motor abnormalities (GMA) can be observed in up to 80% of all patients with schizophrenia and in 66% of first-episode, antipsychotic-naive patients [1–5]. To a lesser degree, they have been observed in individuals considered at ultra-high risk (UHR) and in unaffected first-degree relatives with a genetic risk for schizophrenia [3, 6, 7]. Some researchers accordingly consider GMA a prognostic biomarker for neurodevelopmental alterations contributing to a vulnerability to the illness [3, 8]. However, acquiring a comprehensive overview of GMA related to schizophrenia is difficult. Descriptions and categorizations vary largely with the conceptual framework and the assessment means of the respective researchers [3, 9–12]. Hirjak et al. [3] for example, categorize four groups of GMA: (a) neurological soft signs (NSS)—externally observable impairments in sensory integration, motor coordination, balance, and sequencing of complex motor acts [1, 13], (b) hyperkinetic abnormal involuntary movements (AIMS), such as dyskinesia, dystonia, akathisia or hyperkinesia, (c) hypokinetic AIMS, such as spontaneous parkinsonism, and (4) catatonic phenomena, which can present as a hyperkinetic (e.g. mannerisms, stereotypy) or a hypokinetic (e.g. stupor, rigidity, immobility) movement disorder.

Pavlidou and Walter [9] in turn, name six distinct categories of GMA: (a) Dyskinesia—AIMS, (b) parkinsonism (c) akathisia—including restlessness and inner tension, (d) NSS (e) catatonia, and (f) psychomotor slowing, affecting fine and gross movements, such as writing or walking. The lack of conceptual clarity also applies to GMA rating scales which, additionally, rely on raters’ subjective observation. They are thus prone to observer bias, depend on rater training for accuracy and are not designed to detect subclinical abnormalities [4, 6, 14–19].

The most established neurobiological findings on GMA originate from studies on NSS [3, 10, 20]. Besides being a sign for the risk of developing schizophrenia (trait factors), they can be used to monitor disease progression (state factors) [20, 21]. They are not only related to psychopathological symptoms of schizophrenia [22] but also to poor cognitive and social functioning of patients. Cuesta and colleagues found strong associations of NSS with impaired performance in attention tasks, speed of processing, verbal and visual memory in first-episode patients [23, 24]. The most frequently reported NSS category in patients with schizophrenia is motor incoordination, comprising the inability to perform rapid alternating movements and difficulties in simple coordination tasks, such as the tandem walk or finger-nose tapping [25–27]. Impaired motor or interlimb coordination has been found to discriminate best between high-risk children and controls, and between patients with schizophrenia or a mood disorder [26, 27].

Recently formed task forces, such as the European collaboration on movement and sensorimotor/psychomotor functioning in schizophrenia and other psychoses (ECSP), attempt a consensus on GMA definitions and underline the great advantages (e.g. sensitivity, linearly related results) of an increased implementation of instrumental assessment [6, 19]. Additionally, researchers from different academic backgrounds have begun experimenting with modern technology to create innovative paradigms for the systematic assessment of GMA in schizophrenia. They include accelerometers in smartphones to study tremor, pressure sensitive foot switches for step analysis, or actigraphy to assess restlessness and overall activity of individuals [28–39]. Despite disturbances in interlimb and motor coordination being one of the motor symptoms most specific to schizophrenia, most studies focus on fine motor performance or movement of the upper limbs [10, 39]. Very few studies examine full-body movement, and if they do, they analyze highly reduced (stride length, cadence) or very broad (overall activity) variables [39–41]. The most detailed assessment of human motion has been done with motion capture (MoCap) technologies [42], showing that the mere movement qualities of anonymized walkers (abstracted to point-light displays) reveal information about their gender, age and affective state [43–47]. To our knowledge, within the psychiatric context full-body MoCap has only been applied in one study to analyze movement patterns with relevance to diagnostics: Michalak et al. [48] compared gait patterns of patients with depression to controls and found a reduced walking speed, arm swing, vertical movement, a slumped posture of the upper body, and an increased lateral sway in patients. Effect sizes ranged between *d* = 0.8 and 1.3.

Taken together, despite various instrumental attempts to quantify GMA [18, 28–39], current diagnostics fail to systematically include the objective evaluation of subtle and overt motor behavior [10]. Available assessment means do not analyze detailed full-body movement or interlimb coordination. Hence, with our study, we aimed at

- piloting an assessment protocol, which allows for a detailed, three-dimensional, full-body gait analysis, and
- defining theory-independent full-body movement markers (MM) for schizophrenia.

To navigate around the lack of conceptual clarity regarding GMA and to facilitate a truly objective assessment, we chose a data-driven approach for the first step, and only in the second step related its results to existing symptom definitions. We are not aware of any other study on schizophrenia applying such an approach. The following hypotheses were addressed:

**H1** The mere MoCap data will reveal significantly different movement characteristics for patients and controls, from which full-body Movement Markers (MM) can be extracted by controlling for confounding variables (medication load, weight).

**H2** Full-body MM are similar to but expand the movement characteristics of individuals with depression found by Michalak et al. [48].

**H3** Particularly interlimb coordination is affected.

**H4** Pronounced MM are associated with pronounced NSS (especially subscale motor coordination, and sensory integration).

**H5** Patients with pronounced negative symptoms display pronounced MM.

## Methods

The study was conducted as part of the collaborative research project “Schizophrenia and the Moving Body” [Center for Psychosocial Medicine (CPM), Heidelberg Center for Motion Research (HCMR), BioMotionLab]. It was embedded in a series of studies on movement of individuals with schizophrenia and conducted in accordance with the declaration of Helsinki [49]. The ethics committee of Heidelberg University’s Medical Faculty approved the study before recruitment start.

### Recruitment procedure

Participants with a diagnosis of schizophrenia were consecutively (2019–2020) recruited from one of four wards (three in-patient, one out-patient ward) of the CPM. Included patients were (1) able to consent, (2) between 18 and 60 years old, (3) diagnosed with a schizophrenia spectrum disorder (ICD-10: F20.0-F20.9) prior to study inclusion by senior psychiatrists unrelated to the study and (4) stable on antipsychotic medication for at least 2 weeks. Exclusion criteria were: (1) acute psychosis (ICD-10: F23), (2) diagnosis with a catatonic or schizoaffective subtype (ICD-10: F20.2, F25.0-F25.9), (3) history of brain trauma, neurological or internal diseases, heavy fractions or prostheses (4) visible tremor, (5) strong visual impairment (6) alcohol/substance abuse or dependency within the past 12 months or a substance-induced psychosis (ICD-10: F19.5), (7) an IQ < 70, (8) an SAS score above 4, (9) pronounced language barriers. Controls were recruited through postings and the University’s website. Exclusion criteria resembled the patients’ ones with one addition: history of psychosis or schizophrenia, personal or in first-degree relatives. All participants gave informed consent prior to participation, were clinically assessed at the CPM and then motion captured at HCMR. A priori power analyses (g*power) suggested a total sample size between 23 and 55 for the detection of medium to large effects (*d* = 0.5–0.8), when assuming an alpha-level of *p* < 0.05. Because previous studies [48] found large effect sizes, we targeted a sample size of at least 40 participants.

#### Clinical assessment

Patients were assessed with the Positive and Negative Syndrome Scale (PANSS) [50], the Heidelberger NSS Scale [17], the Brief Psychiatric Rating Scale (BPRS) [51], and the Simpson-Angus Scale (SAS) [52, 53] (parkinsonoid). Controls were assessed with the Heidelberger NSS Scale.

#### Movement assessment

Lab equipment and functionality were explained to prevent psychotic triggers. A set of 49 infrared-reflective markers was attached to the participants skin and skintight sportswear (see C-Motion [54] for the detailed marker set). 8 Oqus500 cameras (Qualisys, Goeteborg, Sweden) tracked participants’ movements. An additional fixed video camera filmed the experiment. Participants were requested to walk back and forth on a path (7 m × 0.70 m) marked with white tape. They performed a series of other movement, balance and coordination tasks (details and results are discussed elsewhere). Walking was chosen, because it is a habituated full-body movement not requiring much cognitive attention but a complex interplay of sensory, motor and balance processes, and a fine-tuning of all limbs. To ensure a natural, “un-performed” walk, participants were asked to walk for a while to “find their most comfortable speed” (at least 3 min) before the actual recording began without further notice. At least 50 steps (8 times through the MoCap volume) were recorded.

### Data analysis

Data were first analyzed algorithm-driven and then following a one-factorial, controlled between-group design. We performed three steps of analyis using different software for the various types of data: (1) By matching the groups for certain characteristics, we aimed at minimizing the influence of confounding variables (see Sect. 2.2.1). (2) We then quantified all visible group differences in movement (movement features, see Sect. 2.2.2), and (3) finally defined movement markers for schizophrenia from the pool of movement features (see Sect. 2.2.3).

#### Step 1: Sample characteristics and propensity score matching

Sample characteristics were analyzed and groups matched in R (Version 4.0.2) [55]. Daily medication load was converted into olanzapine equivalents (OPZ) following the classical mean dose method by Leucht and colleagues [56]. To match an equal-sized subgroup of controls to the available patients, we performed propensity score matching with five variables inherently correlated with gait: (a) gender, (b) age, (c) height, (d) weight, (e) BMI. We chose logistic regression for the estimation of propensity scores and created a matched sample using the one-to-one approach [57–59]. Except for the variable gender (exact matching), we chose nearest-neighbor matching. Matching was successful in reducing covariate imbalance for all variables except weight and, consequently, BMI. Hence, we based all further analysis on the matched and reduced sample and controlled for weight within the data-based exploration of movement patterns and the auxiliary analysis. See the supplementary material for details on matching.

#### Step 2: Data-driven analysis of movement patterns (movement features)

**Fig. 1:**
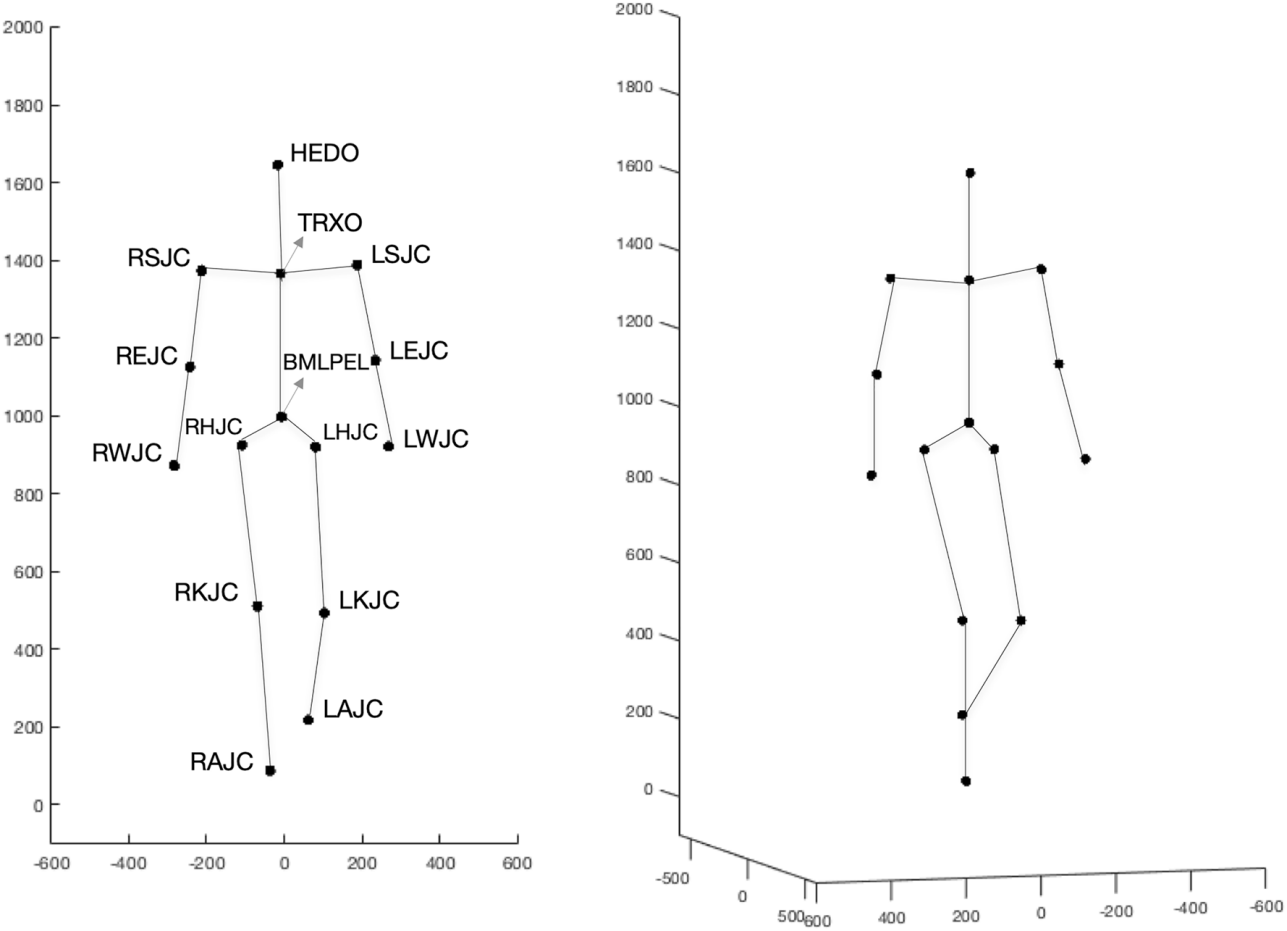
Visualization of the joint centers (JC). They are located at the center of the head (HEDO), the sternum (TRXO), the shoulders (LSJC, RSJC), elbows (LEJC, REJC), wrists (LWJC, RWJC), the center of the pelvis (BMLPEL), hips (LHJC, RHJC), knees (LKJC, RKJC), and the ankles (LAJC, RAJC). The figure displays a film still of the average walker, derived from the entire sample. It can be viewed from the front (left picture) and rotated along all three axes (right picture). It is the basis for the discriminant walker, which is visualized as increments of the average walker. Axes: *x* = walking direction, *y* = lateral direction, *z* = vertical direction

The MoCap data were analyzed with Qualisys Track Manager (Version 2018) and Matlab (Version R2020a). To avoid artifacts in the motion data, first and last centimeters of the walks were excluded from the analysis. For the quantification of movement features, we followed Troje’s [44, 60] computational framework. Due to space limitations, we can only give an overview of the algorithm. See [44] for a detailed description of the single computational steps. First, we computed the locations of 15 joint centers from the 49 marker trajectories. See Fig. 1 for a visualization of the joint center locations and [61–64] for the definition of the bone landmarks. With the help of Fourier Decomposition (FD), the joint center locations were linearized and redundancy was reduced.

We then computed a principal component analysis (PCA) across all Fourier-decomposed walkers to reduce dimensionality of the linear walker space [44, 47] (ten principal components, see [44, p. 10] for the decision on the amount of components of the PCA). To create linear classifiers, we computed a linear discriminant function (LDF), regressing the class indicator (patients, controls) on the walkers’ projections in the low-dimensional Eigenwalker space. To account for group differences in weight, we repeated the LDF computation, regressing the weight on the Eigenwalkers. By multiplying the second LDF (weight) with the transpose of the original LDF (patients, controls), we extracted components that can be explained by weight differences. We subtracted those components from the original LDF. Using the coefficients of the rectified LDF and the Eigenwalkers of the PCA, we created a discriminant walker (DW), an animated visualization of the set of movement patterns that the LDF extracted as classifiers [44]: see https://www.biomotionlab.ca/martin2022/. LDF classifiers, however, remain on data-level, meaning they essentially refer to moving dots in space. To quantify visible movement features, which could be compared statistically, amplitudes and visualizations of the DW were repeatedly examined and rated by different members of the research team. Visible differences were gathered, categorized and computed for each participant on the basis of the FD data for one gait cycle. We aimed at a comprehensive mathematical description of the groups’ dynamic, full-body movement differences (no structural differences: e.g. body size or hip width). Hence, for some movement differences, we propose multiple quantification options (movement features), which either follow Troje or Michalak [44, 48, 65] by capturing body parts’ amount of movement in space, or biomechanical recommendations, by quantifying the extent of motion in a respective joint (“Utilized Range of Motion” (URM)). See Table 1 in the supplementary material for computational details on all movement features.

**Table 1:** Demographics of the matched sample

	Control (*N* = 20)	Patient (*N* = 20)	Total (*N* = 40)
Gender			
Male	14 (70.0%)	14 (70.0%)	28 (70.0%)
Female	6 (30.0%)	6 (30.0%)	12 (30.0%)
Diverse	0 (0%)	0 (0%)	0 (0%)
Age (years)			
Mean (SD)	38.2 (11.1)	39.0 (11.8)	38.6 (11.3)
Weight (kg)			
Mean (SD)	80.7 (15.3)	91.4 (16.1)	86.1 (16.4)
Height (cm)			
Mean (SD)	178 (9.22)	177 (9.87)	177 (9.43)
BMI			
Mean (SD)	25.4 (3.62)	29.0 (3.36)	27.2 (3.89)
Handedness			
Right	18 (90.0%)	17 (85.0%)	35 (87.5%)
Left	2 (10.0%)	3 (15.0%)	5 (12.5%)
Nationality			
German	17 (85.0%)	19 (95.0%)	36 (90.0%)
Other	2 (10.0%)	1 (5.0%)	3 (7.5%)
Many	1 (5.0%)	0 (0%)	1 (2.5%)
Mother tongue			
German	15 (75.0%)	18 (90.0%)	33 (82.5%)
Other	5 (25.0%)	2 (10.0%)	7 (17.5%)
Family status			
Unwed	12 (60.0%)	17 (85.0%)	29 (72.5%)
Married	5 (25.0%)	2 (10.0%)	7 (17.5%)
Widowed	0 (0%)	0 (0%)	0 (0%)
Divorced	3 (15.0%)	1 (5.0%)	4 (10.0%)
Years of education			
Mean (SD)	17.5 (2.61)	14.7 (4.19)	16.3 (3.54)
Missing	0 (0%)	7 (35.0%)	7 (17.5%)
Job			
In training	6 (30.0%)	1 (5.0%)	7 (17.5%)
Employed	11 (55.0%)	7 (35.0%)	18 (45.0%)
Self employed	3 (15.0%)	0 (0%)	3 (7.5%)
Retired	0 (0%)	2 (10.0%)	2 (5.0%)
Unemployed	0 (0%)	7 (35.0%)	7 (17.5%)
On sick leave	0 (0%)	2 (10.0%)	2 (5.0%)
Other	0 (0%)	1 (5.0%)	1 (2.5%)
Olanzapine equivalents			
Mean (SD)	0 (0)	17.2 (8.30)	–
Years of illness			
Mean (SD)	0 (0)	12.6 (11.5)	–
Number of psychoses			
Mean (SD)	0 (0)	5.05 (4.86)	–

Despite propensity score matching, the groups differed significantly concerning their weight and BMI. Covariate balance was ensured by comparing group means and calculating variance ratios. There were no significant group differences in any of the other variables

#### Step 3: Determination of movement markers (MM) and correlations with clinical scales

Group comparisons of movement features and correlations were computed with IBM SPSS (Version 27.0.0.0). To determine MM for schizophrenia, all movement features were tested for significance. First, we computed *t* tests (two-sided, independent groups). We applied Bonferroni correction (*p* < 0.0003 = 0.05/154) for multiple testing but due to the explorative nature of our study also acknowledged initially significant variables not withstanding the correction. We again controlled for the weight of participants in an ANCOVA. In an auxiliary analysis, we assessed the influence of the medication load, by (a) correlating significant movement features with OPZ, and (b) splitting the patient group into a high and low dosage group and comparing significant movement features with second *t* tests. Movement features which proved significant in the main *t* tests and the ANCOVA and non-significant in the auxiliary analysis were defined as MM. Furthermore, we chose movement features which could be summarized for both body sides. Finally, we correlated the defined MM with the clinical scales PANSS, BPRS and NSS. Correlations with PANSS and BPRS, being schizophrenia-specific, were calculated for the patient group only. We correlated the MM with the conventional three factor model of the PANSS (Positive, Negative, Global) [50] as well as with the five-factor model of van der Gaag et. al (Positive, Negative, Disorganized, Excitement, Emotional Distress) [66, 67]. Correlations with NSS were calculated for the entire sample. We correlated the MM with gait specific items of the NSS scale (Station and Gait, Tandem Walk) as well as with its five subscales (Motor Coordination, Sensory Integration, Complex Motor Tasks, Right/Left Spatial Orientation, Hard Signs) [17].

## Results

### Sample characteristics

We screened over 140 and included 50 participants (22 patients, 28 controls). Due to drop out and propensity score matching, we analyzed the data of 40 individuals: 20 patients, 20 controls. See Table 1 for detailed sample characteristics.

### Data-based movement markers

Results of the group comparison are shown in Table 2. Due to space limitations, it only contains movement features, which display significant group differences (see results of all t tests in Table 2 of the supplementary material). Features which withstood Bonferroni correction are marked bold.

- *Basic features* patients and controls walked with significantly different speed (Mean velocity) resulting from a smaller stride or step length, not from a significantly different cadence (CA, SF see Table 2 supplementary material). They differed significantly in modeling power (Mean Power), indicating a less regular walk in the patient group, and varied more in their modeling power across moves (Standard Deviation of Power). It generally seems harder to model patients’ walk with FD.
- Of all postural features (see Tables 1 and 2 in the supplementary material), only the angle between clavicle and head (head angle) was significantly different between the groups. Heads of patients “hang” more than those of controls.
- *Sway of body parts *patients displayed a significantly reduced 3D, horizontal and anterior–posterior (AP) arm sway, a significantly reduced 3D elbow and knee sway, and a significantly increased lateral body sway..
- *Interplay of limb movement (interlimb coordination)* patients displayed a significantly increased ratio of left and right arm or wrist movement, indicating lesser adjustment of the two body sides. Furthermore, we found significant differences in relational movement of the wrists and elbows (Ratio Wrist Elbow, Difference Wrist Elbow). Patients not only move wrists and elbows less in general, but they also move their wrists much less in relation to their own elbows, indicating stiffer arm movements. Similarly, we found significant differences in the relational movement of the arms and legs (Ratio Leg Arm, Difference Leg Arm) and shoulders and hips (Ratio Shoulder Hip, Difference Shoulder Hip). Patients do not adjust arm to leg movement (significantly more leg movement in relation to arm movement) or shoulder and hip movement to each other. Controls use their hips flexibly, patients walk with rather stiff hips, in a pendulum-like manner (twice as much lateral shoulder than hip movement).
- *Utilized range of motion* we found significant differences between the groups of URM in AP and lateral direction, both of the upper and lower, left and right arm (URM Left Arm AP − URM Right Elbow lateral). Analyzing physicality-independent, biomechanical measures, we can confirm the finding that patients generally use upper and lower arms less than controls. Furthermore, the 3D angle inside the elbow changes significantly less for patients within one gait cycle (URM Left and Right Elbow 3D), indicating a rather stiff usage of the arms. We also could replicate significant differences in LBS by looking at changes in the lateral movement of the thorax in relation to the entire body (URM Thorax lateral).
- *Relational URM* calculating the ratio of arm movement in AP and lateral direction, we found that movement in walking direction is more dominant in controls than in patients (Ratio URM Left and Right Arm, Difference URM Left and Right Arm). The strong expression of the control group’s arm movement in walking direction can be interpreted as a goal directedness of the arms or as less “unnecessary” movement in lateral directions. This result is supported by similar group differences concerning the shoulders: patients move their shoulders more in lateral direction, controls move them almost with the same amount in AP and lateral direction (Ratio URM Left and Right Shoulder, Difference URM Left and Right Shoulder). Furthermore, patients show smaller differences in AP and lateral movement of their upper and lower arms. They move their arms in a stiffer or less flexible way (Ratio Left Elbow Left Arm AP − Difference Right Elbow Right Arm lateral). This result replicates and refines the finding of a decreased URM in the elbow joint of patients.
- All sway velocity measures are significantly different between the groups.
- Patients display significantly more pronounced ratios of knee and elbow velocities.

**Table 2:** Significant Results of first t test

Features	*M*		95% CI		*t*_(38)_	*p*	Cohen’s *d*
	Patient (*N* = 20)	Control (*N* = 20)	LL	UL			
a) Basic features							
Mean velocity	1.084	1.209	− 0.223	− 0.027	− 2.571	0.014*	− 0.813
Mean stride length	1.215	1.330	− 0.202	− 0.029	− 2.696	0.010*	− 0.853
Regularity of walk							
Mean power	**0.993**	**0.995**	**− 0.002**	**− 0.001**	**− 3.971**	0.000***	**− 1.256**
Standard deviation of power	0.0008	0.0005	0.0001	0.0003	2.899	0.006**	0.917
b) Postural features							
Head angle	**12.867**	**5.301**	**3.854**	**11.274**	**4.127**	0.000**	**1.309**
c) Sway of body parts							
Wrist sway left 3D	**261.246**	**418.665**	**− 240.747**	**− 74.091**	**− 3.824**	0.000***	**− 1.209**
Wrist sway right 3D	**217.250**	**384.860**	**− 240.966**	**− 73.872**	**− 4.652**	0.000***	**− 1.471**
Arm sway 3D	**239.248**	**401.762**	**− 234.735**	**− 90.294**	**− 4.555**	0.000***	**− 1.441**
Wrist sway left horizontal	247.086	396.892	− 230.792	− 68.818	− 3.745	0.001**	− 1.184
Wrist sway right horizontal	**203.823**	**366.547**	**− 234.778**	**− 90.670**	**− 4.572**	0.000***	**− 1.446**
Arm sway horizontal	**225.55**	**381.719**	**− 226.952**	**− 85.577**	**− 4.475**	0.000***	**− 1.415**
Arm sway left AP	**240.210**	**392.976**	**− 232.807**	**− 72.724**	**− 3.864**	0.000***	**− 1.222**
Arm sway right AP	**169.308**	**363.490**	**− 239.992**	**− 94.371**	**− 4.648**	0.000***	**− 1.470**
Arm sway AP	**218.259**	**378.233**	**− 230.592**	**− 89.355**	**− 4.586**	0.000***	**− 1.450**
Elbow sway left 3D	141.424	202.882	− 95.531	− 27.385	− 3.651	0.001**	− 1.155
Elbow sway right 3D	**126.004**	**185.913**	**− 88.892**	**− 30.926**	**− 4.185**	0.000***	**− 1.323**
Elbow sway 3D	**133.714**	**194.397**	**− 90.415**	**− 30.952**	**− 4.132**	0.000***	**− 1.307**
Lateral body sway	41.841	32.612	3.143	15.317	3.070	0.004**	0.971
Knee sway left 3D	299.171	323.829	− 48.540	− 0.776	− 2.090	0.043*	− 0.661
Knee sway right 3D	289.709	321.029	− 55.098	− 7.541	− 2.666	0.011**	− 0.843
Knee sway 3D	294.440	322.429	− 51.227	− 4.751	− 2.438	0.02*	− 0.771
d) Relational sway of body parts							
Relation of body sides							
Ratio wrist sway left right AP	1.753	1.255	0.0176	0.979	2.098	0.043*	0.664
Relation of body parts							
Ratio left wrist elbow	1.781	2.063	− 0.502	− 0.062	− 2.595	0.013*	− 0.821
Difference left wrist elbow	119.822	215.783	− 149.110	− 42.811	− 3.655	0.001**	− 1.156
Ratio right wrist elbow	1.663	2.054	− 0.625	− 0.156	− 3.375	0.002**	− 1.067
Difference right wrist elbow	**91.246**	**198.947**	**− 155.360**	**− 60.041**	**− 4.575**	0.000***	**− 1.447**
Ratio wrist elbow	1.738	2.062	− 0.526	− 0.123	− 3.260	0.002**	− 1.031
Diff wrist elbow	**105.534**	**207.365**	**− 147.872**	**− 55.789**	**− 4.477**	0.000***	**− 1.416**
Ratio leg arm	3.479	1.929	0.379	2.721	2.679	0.011*	0.847
Difference leg arm	394.516	282.596	39.225	184.616	3.117	0.003**	0.986
Ratio shoulder hip	1.878	1.172	0.170	1.241	2.666	0.011*	0.843
Difference shoulder hip	15.437	1.007	4.931	23.929	3.075	0.004**	0.972
(e) Utilized range of motion (URM)							
URM left arm AP	9.753	15.027	− 8.276	− 2.271	− 3.556	0.001**	− 1.124
URM right arm AP	**8.641**	**13.647**	**− 7.538**	**− 2.473**	**− 4.001**	0.000***	**− 1.265**
URM right arm lateral	1.529	2.043	− 0.880	− 0.147	− 2.835	0.007**	− 0.896
URM left elbow AP	19.342	30.572	− 17.228	− 5.231	− 3.790	0.001**	− 1.199
URM right elbow AP	**15.601**	**28.653**	**− 18.722**	**− 7.381**	**− 4.660**	0.000***	**− 1.474**
URM left elbow lateral	3.617	6.254	− 4.310	− 0.964	− 3.191	0.003**	− 1.009
URM right elbow lateral	**2.154**	**5.072**	**− 4.103**	**− 1.732**	**− 4.980**	0.000***	**− 1.575**
URM left elbow 3D	3.988	6.485	− 4.339	− 0.654	− 2.743	0.009**	− 0.867
URM right elbow 3D	3.618	6.956	− 5.251	− 1.425	− 3.533	0.001**	− 1.117
URM thorax lateral	1.964	1.559	0.128	0.681	2.965	0.005**	0.938
URM left shoulder lateral	1.972	1.656	0.001	0.631	2.032	0.049*	0.643
URM right shoulder lateral	1.958	1.590	0.053	0.682	2.369	0.023*	.749
URM left shoulder AP	**1.308**	**1.757**	**− 0.669**	**− 0.229**	**− 4.127**	0.000**	**− 1.305**
URM right shoulder vertical	.394	0.298	0.002	0.189	2.057	0.047*	.651
URM right hip 2D	1.197	1.584	− 0.748	− 0.026	− 2.168	0.037*	− .686
URM right hip AP	3.506	4.487	− 1.864	− 0.097	− 2.247	0.031*	− .711
URM left hip vertical	.351	0.437	− 0.171	− 0.002	− 2.067	0.046*	− .654
f) Relational utilized range of motion (URM)							
Relation of movement in AP and lateral direction of the same body part							
Ratio URM left arm	4.518	6.212	− 3.083	− 0.303	− 2.466	0.018*	− .780
Diff URM left arm	7.406	12.466	− 7.820	− 2.300	− 3.712	0.001**	− 1.174
Diff URM right arm	7.112	11.604	− 7.0166	− 1.967	− 3.602	0.001**	− 1.139
Diff URM left elbow	15.725	24.318	− 13.393	− 3.792	− 3.624	0.001**	− 1.146
Ratio URM left shoulder	**0.685**	**1.152**	**− 0.668**	**− 0.266**	**− 4.704**	0.000***	**− 1.488**
Diff URM left shoulder	**− 0.664**	**0.101**	**− 1.090**	**− 0.441**	**− 4.776**	0.000***	**− 1.510**
Ratio URM right shoulder	0.867	1.330	− 0.729	− 0.197	− 3.520	0.001**	− 1.113
Diff URM right shoulder	− 0.262	0.337	− 0.966	− 0.231	− 3.302	0.002**	− 1.044
Relation of movement of different joints							
Difference left elbow left arm AP	9.589	15.545	− 9.441	− 2.471	− 3.459	0.001**	− 1.094
Ratio left elbow left arm lateral	**1.502**	**2.468**	**− 1.459**	**− 0.472**	**− 3.964**	0.000***	**− 1.253**
Difference left elbow left arm lateral	1.270	3.693	− 3.719	− 1.128	− 3.787	0.001**	− 1.198
Ratio right elbow right arm AP	1.717	2.125	− 0.672	− 0.145	− 3.136	0.003**	− .992
Difference right elbow right arm AP	**6.960**	**15.006**	**− 11.674**	**− 4.418**	**− 4.490**	0.000***	**− 1.420**
Ratio right elbow right arm lateral	1.459	2.625	− 1.809	− 0.523	− 3.673	0.001**	− 1.161
Difference right elbow right arm lateral	**0.625**	**3.029**	**− 3.479**	**− 1.329**	**− 4.528**	0.000***	**− 1.432**
Difference left shoulder hip lateral	− 1.247	− 2.424	0.126	2.226	2.267	0.029*	0.717
Difference right shoulder hip lateral	− 1.169	− 2.329	0.075	2.244	2.165	0.037*	0.685
Ratio left shoulder hip vertical	1.393	0.814	0.080	1.077	2.350	0.024*	0.743
Difference left shoulder hip vertical	0.055	− 0.118	0.045	0.302	2.724	0.010*	0.862
Ratio right shoulder hip vertical	1.352	0.804	0.005	1.092	2.041	0.048*	0.646
Difference right shoulder hip vertical	0.029	− 0.120	0.018	0.279	2.303	0.027*	0.728
g) Velocities of body parts							
Velocity left wrist 2D	222.366	362.129	− 216.566	− 62.960	− 3.684	0.001**	− 1.165
Velocity right wrist 2D	**182.898**	**333.890**	**− 218.867**	**− 83.116**	**− 4.503**	0.000***	**− 1.424**
Velocity left wrist 3D	235.064	382.036	− 226.148	− 67.795	− 3.758	0.001**	− 1.188
Velocity right wrist 3D	**194.879**	**350.584**	**− 224.663**	**− 86.747**	**− 4.571**	0.000***	**− 1.445**
Velocity left elbow 3D	126.523	184.208	− 89.630	− 25.741	− 3.656	0.001**	− 1.156
Velocity right elbow 3D	**112.640**	**168.498**	**− 82.801**	**− 28.917**	**− 4.197**	0.000***	**− 1.327**
Velocity left knee 3D	266.733	294.250	− 53.233	− 1.800	− 2.166	0.037*	− .685
Velocity right knee 3D	258.404	291.409	− 58.005	− 8.005	− 2.673	0.011*	− .845
h) Relational velocities of body parts							
Ratio velocity left knee elbow	2.244	1.731	0.156	0.870	2.908	0.006**	.919
Ratio velocity right knee elbow	2.482	1.846	0.224	1.047	3.126	0.003**	.988

^*^ *p* < 0.05*,* ** *p* < 0.01, ***p < 0.001. LL = Lower Level, UL = Upper Level, AP = anterior–posterior, 3D = 3 dimensional, 2D = horizontal

Definitions and calculations of the movement features are shown in Table 1 in the supplementary material. All t-tests (also non-significant results) can be viewed in the supplementary material (Table 2). We applied Bonferroni adjustment for multiple testing: p < 0.0003 = 0.05/154. Results, which remained significant after Bonferroni adjustment are marked bold

Effect sizes are considerably large (*d* = 0.6–1.5). None of the auxiliary correlations and *t* tests were statistically significant (see Tables 3 and 4 in the supplementary material) indicating that movement features are indeed independent of medication. Applying the definition rules mentioned above (see also the auxiliary ANCOVA—Table 5 in the supplementary material), we defined 16 full-body MM for schizophrenia. Table 3 summarizes the MM, their manifestation within the groups and ways to quantify them (movement features). Except for a decreased vertical body sway, we found similar MM in patients with schizophrenia, which Michalak and colleagues [48] found in individuals with depression. Additionally, we found a reduced step length, a reduced regularity of gait and various MM indicating a reduced ability to integrate the movement of body sides and limbs. Following Bonferroni adjustment, significance was maintained for the regularity of the gait, the arm and elbow sway, the flexibility of arm movement, the goal directedness of the shoulder movement and arm and elbow sway velocities. Hence, we can confirm H1 to H3.

**Table 3:** Movement markers of schizophrenia

Movement marker	Quantification (movement feature acronym)	Manifestation in people with schizophrenia and controls	Relation to neurological soft signs
*Postural markers*			
Head posture	Angle between head and clavicle. A bigger angle: more slumped posture/more hanging head (alphaHEAD)	Patients display significant bigger angles than controls, indicating their head is “hanging” more than the one of controls. Their posture is more slumped	A slumped posture is positively correlated with the amount of NSS, especially with the subscales Motor Coordination and Sensory Integration. The higher the amount of NSS, the more slumped the posture of the participants
*Kinematic markers*			
Regularity/periodicity of gait	Modeling an individual gait with FD requires the walk to be periodic and repetitive. To quantify the periodicity of the groups’ gait, we computed the participants’ modeling power, a measure for the average variance covered by the number of Fourier components in the chosen model (in our case 2). Non-periodic or irregular elements are reflected in a lower power of the FD. A higher power indicates a more periodic or rhythmic walk. (Mpower)	Patients display a significantly lower mean modeling power than controls. It is harder to model their gait with periodic Fourier decomposition. Their walk is less regular than the ones of controls	The gait regularity is negatively correlated with the amount of NSS (subscales Motor Coordination, Sensory Integration, Complex Motor Tasks, Hard signs). The higher the amount of NSS, the more irregular the walk
Variation of regularity/periodicity of gait	Variation of the gait regularity within one walk: Standard Deviation of Average Power (see MPower) across 8 moves (SDPower)	Patients display a higher variation in their gait regularity. While the moves of controls can be modeled equally well, the moves of patients vary in their modeling power	The variation of the gait regularity is positively correlated with the amount of NSS (subscales Motor Coordination, Sensory Integration, Complex Motor Tasks)
Stride length	The average length of one stride (two steps) (MSL, MSTRL)	Patients walk with smaller strides and make significantly smaller steps	The step length is negatively correlated with the amount of NSS (subscale Motor Coordination, Sensory Integration, Right/Left Spatial Orientation). The higher the amount of NSS, the shorter the steps
Arm sway	3D/horizontal/forward Wrist Sway of left and right side and averaged across sides (AS3, WSL3, WSR3*,* AS2, WSL2, WSR2*,* AS1x, ASL1x, ASR1x) *or* changes in arm pit angle (URMLAx, URMRAx)	Patients display a lower arm sway. On average they move their arms less than controls. This applies to all quantifications of arm sway (3D, horizontal, AP, physicality independent)	Arm sway is negatively correlated with NSS (subscales Motor Coordination, Sensory Integration, Complex Motor Tasks). The higher the amount of NSS, the smaller the amount of arm movement. *Furthermore, there is a positive correlation with the subscale hard signs. This somewhat contradicts the other correlation patterns*
Elbow sway	3D Elbow Sway of left and right side and averaged across sides (ES3, ESL3, ESR3) or Changes of elbow joint angle (3D: URMLE3, URMRE3, AP: URMLEx, URMREx, or lateral: URMLEy, URMREy)	Patients display a lower elbow sway than controls. On average they move their elbows less than controls. Furthermore they display less movement in their lower arm, in all directions	Elbow sway is negatively correlated with NSS, especially with the subscales Motor Coordination, Sensory Integration and Complex Motor Tasks. The higher the amount of e.g. Motor coordination Deficits the smaller the amount of elbow/lower arm movement. *Like the arm sway 3D elbow sway is positevly correlated with the subscale Hard Signs*
Knee sway	3D Knee Sway of left and right side and averaged across sides (KS3, KSL3, KSR3)	While walking, patients move their knees less in all directions than controls	*We could not find a significant correlation with NSS*
Lateral body sway	Mean amplitude of left and right shoulder in lateral direction (LBS) or changes of Thorax angle in lateral direction (URMTRy)	Patients display a larger lateral sway, lateral movement of the upper body while walking	Lateral body sway (LBS) is positively correlated with NSS subscale Complex Motor Tasks, indicating a higher amount of difficulties with complex motor tasks when displaying more lateral sway while walking
*Velocity markers*			
Gait velocity	Average speed across all 8 moves (MV)	Patients walk with less speed than controls	The average speed is negatively correlated with the amount of NSS (subscale Motor Coordination). The higher the amount of NSS, the slower the walk
Arm sway velocity	Wrist Sway multiplied by Cadence (3D: vWSL3, vWSR3, Horizontal: vWSL2, vWSR2)	Although patients don’t walk with a lower cadence, they display a slower arm sway than controls for both sides (left and right)	The velocity of the arm movement is negatively correlated with NSS, subscale Motor Coordination, and overall NSS. The faster the movement, the smaller motor coordination deficits
Elbow sway velocity	3D Elbow Sway multiplied by Cadence (vESL3, vESR3)	Patients display a slower 3D elbow sway than controls for both sides (left and right)	The velocity of the elbow movement is negatively correlated with NSS, subscale Motor Coordination, and overall NSS. The faster the movement, the smaller motor coordination deficits
*Relational markers (interlimb coordination)*			
Adjustment of body sides (left/right)	Ratio of wrist sway on left and right side in AP direction: A bigger ratio indicates a higher difference between the sides, one side moves more than the other (RatioWSLR1)	Patients display a higher ratio, indicating that one arm moves more than the other. The Ratio of controls is closer to 1, indicating that arms on both body sides are moved with the same amount	The adjustment of body sides is positively correlated with the amount of NSS, especially Motor Coordination. The higher the ratio of the arms (the harder the adjustment), the bigger the amount NSS
Goal directedness of movement	Difference of URM of Left/right upper arm (diffURMLA, diffURMRA) or shoulder (diffURMLS, ratioURMLS, difffURMRS, ratioURMRS) in AP and lateral direction	Patients display less movement in AP direction of the arms when related to movement in lateral direction. Patients also display more lateral movement of the shoulders, when related to AP movement, Controls move their shoulders more in AP or walking direction. Patients’ movement seems less goal or forward directed. *The same pattern can be found for the lower Arms (e.g. diffURMLE)*	The goal directedness of the arm and shoulder movement is negatively correlated with the amount of NSS, especially Motor Coordination. The more goal directed the movement, the smaller the amount of NSS
Flexibility of limb movement	Ratio/Difference of mean wrist and elbow sway of both sides (left/right) all directions (RatioWEd, DiffWEd) or difference of URM of left upper arm (arm) and left lower arm (elbow) in AP or lateral direction (diffLELAx, diffRERAx, diffLELAy, diffRERAy)	All Participants move their lower arms/wrirsts more than their upper arms/elbows but patients display less lower arm movement in relation to their upper arm movement. Their arm movement while walking is more rigid. A look at the physicality-independent flexibility measures reveals that stiffness is apparent in both, AP and lateral movement of the arms	The flexibility of the arm movement is negatively correlated with the amount of NSS (subscale Motor Coordination, Sensory Integration and Complex Motor Tasks). A higher flexibility is related to a smaller amount of NSS
Adjustment/integration of limb movement	Ratio of leg and arm movement (RatioLAd, DiffLAd) or difference of shoulder and hip movement (DiffSHd). A higher ratio/difference indicates less adjustment of arm to leg or shoulder to hip movement	Patients move their legs much more in relation to their arms than controls or their arms much less in relation to their legs. They don’t adjust arm movement to steps. Furthermore, patients display less of an adjustment of shoulder and hip movement	The adjustment of leg and arm movement and of shoulder and hip movement is positively correlated with the amount of NSS, especially Motor coordination. More adjustment of arm and leg or shoulder and hip movement (smaller ratio) is related to a smaller amount of NSS
Adjustment/integration of sway velocities	Ratio of velocity of Elbow and Knee Sway (ratiovLKE3, ratiovRKE3). A smaller ratio indicates a better adjustment of sway velocities	Patients and controls display differences in the adjustment of body part velocities. They move their knees 2.24 times as fast as their elbows, controls only 1.7 times as fast. This is in line with the fact that arms are used much less and in a stiffer way	The adjustment of sway velocities between arms and legs is positively correlated with the amount of Motor Coordination Deficits and the amount of overall NSS, but negatively correlated with hard signs. The better the adjustment, the smaller the motor coordination deficits but the more pronounced the hard signs

One “move” is one way through the MoCap volume in the HCMR lab. We recorded 8 moves for each participant. The column “Movement Marker” (MM) refers to the concepts, which point toward schizophrenia. Due to the exploratory nature of our study, we chose to ignore p-value correction for the determination of MM. The column “Quantification” offers options of quantification that we found for the respective markers. It also displays the shortages of the feature names, which can be used to look up details of all defined features and their quantification in Table 1 in the supplementary material and (upon request of the first author) in the respective Matlab script

*AP* anterior–posterior

**Table 4:** Correlations of movement markers with clinical symptom load

Movement markers								
	Gait velocity (MV)	Stride length (MSTRL)	Head posture (alphaHEAD)	Regularity of gait (MPower)	Variation of gait regularity (SDPower)	Arm sway (AS3)	Elbow sway (ES3)	Knee sway (KS3)
*Clinical Scales*								
PANSS: pos	− 0.192	− 0.255	0.326	− 0.411	0.288	− 0.352	− 0.316	− 0.233
PANSS: neg	0.024	− 0.111	0.229	− 0.304	0.257	− 0.393	− 0.282	− 0.165
PANSS: glob	− 0.177	− 0.140	0.178	− 0.307	0.332	− 0.356	− 0.196	− 0.206
VDG: positive	− 0.210	− 0.173	0.147	− 0.411	0.233	− 0.346	− 0.283	− 0.198
VDG: negative	− 0.024	− 0.143	0.247	− 0.271	0.324	− 0.399	− 0.301	− 0.177
VDG: disorganisation	− 0.032	− 0.010	0.412	− 0.346	0.331	− 0.336	− 0.187	− 0.008
VDG: excitement	− 0.247	− 0.179	0.161	− 0.389	0.208	− 0.388	− 0.304	− 0.180
VDG: emotional distress	− 0.145	− 0.255	− 0.004	− 0.270	0.179	− 0.266	− 0.147	− 0.339
PANSS: total	− 0.117	− 0.165	0.243	− 0.349	0.314	− 0.390	− 0.268	− 0.210
BPRS	− 0.292	− 0.323	0.112	− 0.276	0.333	− 0.288	− 0.193	− 0.366
NSS: station and gait	− 0.402**	− 0.441***	0.567***	− 0.518***	0.474**	− 0.622***	− 0.564***	− 0.366*
NSS: tandem walk	− 0.186	− 0.164	0.511***	− 0.275	0.377*	− 0.405*	− 0.327*	− 0.078
NSS1: motor coordination	− 0.303	− 0.307	0.370*	− 0.425**	0.349*	− 0.528**	− 0.439**	− 0.286
NSS2: sensory integration	− 0.294	− 0.343*	0.499**	− 0.325*	0.472**	− 0.443**	− 0.371*	− 0.228
NSS3: complex motor tasks	− 0.147	− 0.131	0.231	− 0.351*	0.329*	− 0.380*	− 0.332*	− 0.117
NSS4: R/L spatial orient	− 0.181	− 0.375*	0.060	− 0.013	0.023	− 0.147	− 0.198	− 0.254
NSS5: hard signs	0.064	0.197	− 0.189	0.336*	− 0.200	0.292	0.337*	0.199
NSS: total	− 0.319*	− 0.358*	0.394*	− 0.387*	0.389*	− 0.516**	− 0.440**	− 0.288

	Lateral body sway (LBS)	Adjustment of body sides (RatioWSLR)	Goal directedness of movement (diffURMLS)	Flexibility of limb movement (RatioWEd)	Adjustment of limb movement (RatioLAd)	Arm sway velocity (vWSL3)	Elbow sway velocity (vESL3)	Adjustment of sway velocity (ratiovLKE)
PANSS: Pos	− 0.148	− 0.382	0.281	− 0.220	− 0.088	− 0.346	− 0.276	0.041
PANSS: Neg	− 0.317	− 0.315	0.381	− 0.375	0.018	− 0.304	− 0.190	0.058
PANSS: Glob	− 0.147	− 0.234	0.372	− 0.394	0.019	− 0.348	− 0.210	− 0.017
VDG: positive	− 0.072	− 0.354	0.259	− 0.235	− 0.082	− 0.394	− 0.315	0.043
VDG: negative	− 0.292	− 0.318	0.402	− 0.369	0.006	− 0.320	− 0.209	0.051
VDG: disorganisation	− 0.282	− 0.272	0.497*	− 0.386	0.021	− 0.346	− 0.217	0.091
VDG: excitement	− 0.052	− 0.147	0.239	− 0.350	0.124	− 0.387	− 0.296	0.071
VDG: emotional distress	− 0.042	− 0.225	0.135	− 0.260	− 0.008	− 0.212	− 0.093	− 0.118
PANSS: total	− 0.218	− 0.313	0.376	− 0.369	− 0.006	− 0.352	− 0.231	0.023
BPRS	− 0.123	− 0.370	0.353	− 0.243	− 0.124	− 0.309	− 0.210	− 0.100
NSS: station and gait	0.198	0.361*	− 0.267	− 0.573***	0.433**	− 0.491**	− 0.451**	0.436**
NSS: tandem walk	0.151	0.215	− 0.239	− 0.451**	0.266	− 0.324*	− 0.285	0.307
NSS1: motor coordination	0.228	0.405*	− 0.456**	− 0.548**	0.435**	− 0.407*	− 0.362*	0.376*
NSS2: sensory integration	0.187	0.269	− 0.168	− 0.464**	0.258	− 0.293	− 0.261	0.234
NSS3: complex motor tasks	0.337*	0.198	− 0.471**	− 0.371*	0.248	− 0.255	− 0.247	0.237
NSS4: R/L spatial orient	− 0.287	0.022	− 0.057	0.002	0.050	− 0.090	− 0.150	0.152
NSS5: hard signs	0.269	− 0.021	− 0.144	0.105	− 0.191	0.304	0.300	− 0.334*
NSS: total	0.228	0.356*	− 0.449**	− 0.518**	0.372*	− 0.364*	− 0.340*	0.336*

Correlations follow Pearson and are two-tailed. Correlations of PANSS and BPRS were calculated for the patient group only, correlations of NSS were calculated with the entire sample. For space-saving reasons, for each MM one quantification option (movement feature acronym) was chosen. NSS1 to NSS5 are subscales of the Heidelberger NSS Scale. “Station and Gait” as well as “Tandem Walk” are two items of the respective scale which are directly related to walking

*VDG* van der Gaag, *Orient*. Orientation

Significant correlations are marked bold

*p < 0.05, **p < 0.01, ***p < 0.001

### Correlations with clinical scales

Table 4 displays the correlations of the MM with the clinical scales. We can confirm H4. Almost all MM (except Lateral Body Sway and Knee Sway) are significantly correlated with total NSS scores and especially with the subscales *Motor Coordination, Sensory Integration* and *Complex Motor Tasks.* The correlations indicate that a stronger manifestation of the respective MM is associated with a stronger manifestation of NSS in general, specifically with coordination and integration related NSS. Furthermore, all objectively measured MM (except Lateral Body Sway and Goal Directedness of Movement) are significantly related to the subjectively rated NSS item “Station and Gait”. Again, and in all cases, a stronger manifestation of the respective MM is related to more disturbances in station and gait. Contrary to this, only some specific MM are correlated with the NSS item “Tandem Walk”: Head Posture, Variation of Gait Regularity, Arm and Elbow Sway, Flexibility of Limb Movement and Arm Sway Velocity.

We can not confirm H5: none of the correlation patterns with the positive, negative, global (PANSS) and overall symptom load (BPRS) are significant. This holds true when looking at van der Gaag’s five-factor model of the PANSS [67]: Except for the MM Goal directedness, which is positively related to the patients’ disorganisation, none of the correlations become significant. The positive correlation of the patients’ goal directedness with the disorganisation subscore contradicts the correlation patterns with NSS scores indicating a stronger manifestation of disorganisation when movements are more goal directed. Some MM display a correlational trend with the PANSS and BPRS. e.g. in the sense that less of an arm sway is related to an increase in positive, negative, disorganisational, excitement related and overall symptoms (e.g. *r*_AS3, PANSS:Pos_). Correlations of integration related features with the symptom load do not show a systematic pattern. Furthermore, we could not find a systematic difference between the interaction of the MM with positive or with negative symptoms.

## Discussion

Our study revealed three major results. First, movement features extracted and quantified instrumentally from basic walking are able to differentiate between individuals with a diagnosis of schizophrenia and without. Second, the MM of schizophrenia are mainly related to the integration and adjustment of body sides, limbs or the direction of movement. Third, most of the MM are associated with increased NSS, particularly motor coordination and sensory-motor integration.

Our theory-independent MM are in line with results of the few previous studies analyzing posture and gait disturbances in people with schizophrenia [39–41, 48, 68, 69]: Cristiano et al. [69] identified forward head tilt as the most common postural feature in early and late-stage schizophrenia and found associations of postural changes with disease severity. Similarly, we identified the head posture as only significant posture marker and found associations of an increased “hanging” of the head with higher levels of NSS items “Station and Gait”, “Tandem Walk” and NSS subscales “Motor Coordination” and “Sensory Integration”. Like Putzhammer and colleagues [41], we identified a significantly decreased gait velocity in patients compared to controls, which can be ascribed to a shorter stride length, not to a decreased cadence. Similarly to Lallart and colleagues [40], who compared the stride-to-stride variability of patients and controls in a “walk alone” and in dual-task conditions and found significant differences in the dual-task conditions only, we did not find group differences concerning the variation of cadence in simple walking. An increased intraindividual variability in kinematic indices such as peak velocity and peak acceleration was also found by Jahn et al., who developed a device to analyse repetitive pronation/supination for subtle kinematic changes [70, 71]. Although pronation/supination, which is generally considered to be a typical NSS for schizophrenia [71], refers to fine motor performance of the hands, Jahn’s finding corresponds to our finding of an increased variation in gait regularity across moves (walks through the MoCap volume). Together with Lallart’s finding, it suggests that schizophrenia is characterized not merely by motor retardation but particularly by motor variability [71].

The fact that gait regularity was the only MM in our study which varied across moves might be related to the simplicity of our movement task. A dual-task condition was part of the above-mentioned additional movement tasks and will be analyzed in forthcoming publications. The striking differences of Lallart’s participants’ stride-to-stride variability while dual-tasking raises the assumption that the identified MM might be augmented by a dual-task. Additionally, Boks et al. [26] identified impaired motor coordination as most specific to patients with schizophrenia when comparing them to patients with a mood disorder; and Schiffman and colleagues [27] identified coordination deficits at the age of 10–13 as predictors of schizophrenia spectrum disorder at the age of 31–33. Although Schiffman and Boks as well restricted their analysis to fine motor performance and did not examine full-body movement, their results coincide with the many relational markers and variability differences we found. The relational features are particularly interesting, because they touch on the idea derived from embodied cognition that a disorganized mind is related to a disorganized body in the sense of a missing intrabodily and sensorimotor integration. Within the field of embodied cognition, the search for underlying brain alterations of schizophrenia is complemented by the analysis of interplay between mind, body and environment [72–74]. Schizophrenia is understood as a form of disembodiment, a missing integration and adjustment of sensorimotor loops, which results in an alienation of somatosensory perception, a lack of emotional expression, and a dissolution of the Gestalt units of movement and action [75, 76]. Successive movements (like one step after the other in walking) are described to lose their relatedness, smooth transition and “grace” [77, 78].

Tsakiris et al. [79] review studies on the sense of body-ownership (“It is my body, which is moving”) and the sense of agency (“I control my movements”) as two basic aspects of an embodied self-experience: while multisensory afferent signals suffice to create a sense of body ownership, it takes the integration of efferent motoric (self-initiated movement) and subsequent afferent sensory signals (multisensory integration) to create a sense of agency and finally a coherent experience of one’s own embodied self. On one hand, the fact that most MM are not only correlated with deficits in “Motor Coordination” but also with deficits in “Sensory Integration” can be seen as further evidence for disembodiment or a lack of multisensory integration in the patients [76]. On the other hand, the consistent relations of the MM with NSS total scores, which not only include motor but also sensory NSS, correspond to the transidagnostic nature of NSS and raises the question if our MM are of transdiagnostic value as well. NSS have been observed in other severe neuropsychiatric conditions such as bipolar disorder, Alzheimer disease or HIV-associated cognitive disorder and are understood by some researchers as signs for neurocognitive impairment in general [20, 80, 81]. While the fact that Michalak et al. [48] did not identify movement patterns related to the adjustment of limbs or body sides in patients with depression suggests the possibility of using MM for the prediction and differential diagnosis of schizophrenia, it remains to be tested if and which MM can be found in patients with other neuropsychiatric diagnoses.

Finally, relating the MM to gait specific NSS items allows for a preliminary validation. While almost all MM were associated with the item “station and gait”, only specific MM (Head Posture, Variation of Gait Regularity, Arm and Elbow Sway, Flexibility of Limb Movement and Arm Sway Velocity) were related to the NSS rating of the tandem walk. This makes sense, since the Tandem Walk—other than simple walking—is a highly coordinated, less habituated movement, which requires a straight posture, flexible limb and dextereous arm usage.

### Limitations and future directions

This was an exploratory study with a relatively small sample size. Non-significant correlations of MM with clinical scales might be a consequence. Hertzog [82] reviews the precision of estimates in pilot studies and appraises samples of 10–20 as sufficient for clinical contexts, given the possibility to specify expected group differences in an a priori power analysis. Our sample size was an interdisciplinary compromise taking into account previous studies, power calculations and the availability of the motion lab. The replication of this study with a larger sample might lead to a systematic association of MM with positive and negative symptoms. Another reason for non-systematic relations of MM with PANSS scores could be the ongoing controversy in the literature about which factor model of the PANSS yields the most useful research results [83].

By conducting a data-driven, comprehensive mathematical description of the groups’ dynamic movement differences, we arrived at a very large amount of movement features, which overlap and correlate. To correct for multiple statistical testing we applied the Bonferroni correction. Streiner and Norman [84] discuss different correction types and arrive at the conclusion that the conservative Bonferroni method might lead to an overcorrection in explorative studies which aim at defining promising leads. Hence, we followed the researchers’ advice against correction in the definition of “areas”, in our case MM, that need follow-up in later studies [84]. In future analyses, we aim at a substantiation of the MM by conducting a factor analysis on the movement features and a multiple regression analysis.

Due to uncertainties concerning the reaction of vulnerable participants to the instrumental assessment, we exclusively examined individuals with a stable second-generation antipsychotic. To control for medication, we did auxiliary statistical analyses. Previous studies found no differences regarding GMA in never-medicated individuals and participants taking second-generation antipsychotics [41] and demonstrated that NSS vary in the course of the illness with psychopathological symptoms [21]. This includes a decline under neuroleptic treatment and speaks against an induction of GMA by medication. However, to entirely rule out the influence of medication on the identified MM, it would be beneficial to examine never-medicated or UHR individuals. This, and the longitudinal measurement of MM might answer the question to what extent behavioral MM can serve as predictors of a transition from a prodromal state to an acute psychosis or as indicators of disease progression. Since recent studies suggest that GMA gradually intensify on a continuum from prodrome to acute psychosis and that NSS improve with medication [1, 3, 20, 21] and since we found significant correlations of our MM with NSS, it is highly probable that a similar continuum can be established for the objectively assessed MM of this study and that the MM are primal symptoms of schizophrenia, which are independent of medical side effects.

Future studies should also assess the neuropathology underlying the identified MM. Recent neuroimaging studies support the hypothesis that GMA are linked to a disrupted “cortico-cerebellar-thalamic-cortical circuit” [1, 3, 10, 85]. However, studies on the neurological mechanisms underlying full-body gait disturbances in schizophrenia are scarce. On the way to defining a distinct motor domain for schizophrenia the present study should be expanded with a portable, neurological assessment, ideally guided by the current Mobile brain/Body imaging (MoBi) approach [86].

Finally, to integrate instrumental assessment into daily clinical practice, less expensive MoCap techniques (e.g. Kinect) should be explored. Prior to recruitment, we experimented with and found great bias in tools which base the motion tracking on inertial measurement units. Hence, we decided to establish subtle MM first and then transfer their mathematical and statistical evaluation to less detailed MoCap data. A greater accessibility and comprehensibility of MoCap data and its analysis might serve a systematic integration of motion assessment into clinical practice, generate great amounts of data and provide the missing link of GMA to the patients’ subjective experience.

### Conclusion

Long-standing negligence of the moving body in schizophrenia research has left us with a diagnostic system heavily weighing positive and cognitive symptoms and underestimating motor abnormalities. The systematic and continuous assessment and staging of MM as well as their correlation with self-experience and subjective well-being could substantially improve early and differential diagnosis of schizophrenia. At length, not only diagnostics but also treatment of schizophrenia would benefit from a systematic staging of MM. While various studies underline the overall beneficiary effects of embodied therapies [75, 87–89], their underlying mechanisms are far from clear. With the help of external cues, Putzhammer et al. [41] could dissolve stride length differences between patients and controls. This is highly encouraging evidence for the beneficiary effect of identifying individual MM of patients and treating them with specifically targeted therapy.

## Supplementary Information

Below is the link to the electronic supplementary material.

Supplementary file1 (DOCX 378 KB)

## Abbreviations

GMA: Genuine motor abnormalities
NSS: Neurological soft signs
UHR: Ultra high risk
CPM: Center for Psychosocial Medicine
HMR: Heidelberg center for motion research
MoCap: Motion capture
FD: Fourier decomposition
LDF: Linear discriminant function
MM: Movement markers
MF: Movement features
URM: Utilized range of motion

## Declarations

### Animations

Visualization of discriminant walker: https://www.biomotionlab.ca/martin2022/
