# Supplementary material for: Movement markers of schizophrenia: a detailed analysis of patients’ gait patterns
Source: Eur Arch Psychiatry Clin Neurosci. 2022 Apr 1;272(7):1347–64. doi: 10.1007/s00406-022-01402-y (PMC9508056; doi:10.1007/s00406-022-01402-y)
Supplement: Supplementary file 1 — Supplementary file1 (DOCX 378 KB) [file 406_2022_1402_MOESM1_ESM.docx]

Full-Body Motor Markers of Schizophrenia

Using motion capture and pattern recognition to analyse gait

Supplementary Material

**Lily Martin** ^a,c^, Kevin Stein^b^, Katharina Kubera^c^ , Nikolaus F. Troje^d^ , Thomas Fuchs^c^

a Department of Psychology, Faculty of Behavioural and Cultural Studies, Heidelberg University, Hauptstraße 47–51, 69117 Heidelberg, Germany

b [Optimization, Robotics and Biomechanics](https://typo.iwr.uni-heidelberg.de/groups/orb/home/), ZITI – Institute of Computer Engineering, Heidelberg University, ML 100, 3rd floor, Berliner Str. 45, 69120 Heidelberg, Germany

c Department of General Psychiatry, Centre for Psychosocial Medicine, Academic Medical Center, Heidelberg University, Voßstraße 4, 69115 Heidelberg, German

d BioMotionLab, Department of Biology, Centre for Vision Research, York University, 4700 Keele Street, Toronto, ON, Canada

# 1. Supplementary Results

**Table 1. Movement features: quantifications of visible group differences in movement**

|  | **Abbreviation** | **Name** | **Explanation** | | **Unit** |
| --- | --- | --- | --- | --- | --- |
| **Basic Features** | | | | | |
| Basic features are related to whole body movement. | | | | | |
| 1 | **MV** | Mean Velocity | Average speed across all 8 moves. One move is one way through the MoCap volume in the HCMR lab. We recorded 8 moves for each participant. | | m/s |
| 2 | **SDV** | Standard Deviation Velocity | Standard deviation of velocity across all 8 moves. | | m/s |
| 3 | **CT** | Cycle Time/Stride Time | A cycle or stride is a gait cycle or two steps. CT is the time a subject needs to make two steps. | | s/cycle |
| 4 | **ST** | Step Time | Time a subject needs to make one step. | | s/step |
| 5 | **CA** | Cadence | Cycle Frequency. The amount of gait cycles (1 cycle = 2 steps) per second. | | cycle/s |
| 6 | **SF** | Step Frequency | The amount of steps per second. | | step/s |
| 7 | **SDCA** | Standard Deviation Cadence | Standard deviation of cadence across 8 moves. | | cycle/s |
| 8 | **SDSF** | Standard Deviation Step Frequency | Standard deviation of step frequency across 8 moves. | | step/s |
| 9 | **MSTRL** | Mean Stride Length | The length of one stride or two steps. | | m/cycle |
| 10 | **MSL** | Mean Step Length | The length of one step. | | m/step |
| 11 | **SDSTRL** | Standard Deviation Stride Legth | Standard deviation of stride length across all 8 moves. | | m/cycle |
| 12 | **SDSL** | Standard Deviation Step Length | Standard deviation of step length across all 8 moves. | | m/step |
| **Postural Features** | | | | | |
| Postural features describe the participants’ stance. Taken together, they differentiate an upright from a slumped posture. | | | | | |
| **Upper Body** | | | | | |
| 13 | **alphaHEAD** | Angle between head and clavicle | | Angle between TRXO and HEDO in x direction. Abbreviations and name of axes are explained below. | degree |
| 14 | **alphaBMLPEL** | Angle between thorax and pelvis | | Angle between BMLPEL and TRXO in x direction. A bigger angle stands for a more slumped posture.  The angle becomes negative, when participants walk with a hollow back (Holkreuz). | degree |
| 15 | **alphaTRX** | Angle between shoulders | | Four Quadrant angles of single shoulder joint centres in 2D space put together. A Value over 180: the shoulders point to the back; Values smaller than 180: shoulders point to the front. Smaller numbers below 180: shoulders point more to the front in relation to Torax, posture is more slumped. | degree |
| 16 | **alphaLA** | Armpit Angle of left Arm | | Angle between vertical line, LSJC and LEJC. | degree |
| 17 | **alphaRA** | Armpit Angle of right Arm | | Angle between vertical line, LSJC and LEJC. | degree |
| **Lower Body** | | | | | |
| 18 | **alphaLK** | Angle of left knee | | 3D angle between LHJC, LKJC, LAJC | degree |
| 19 | **alphaRK** | Angle of right knee | | 3D angle between RHJC, RKJC, RAJC | degree |
| **Whole Body** | | | | | |
| 20 | **space** | Space taken in by posture in relation to BMI | | Volume of 3D boundary around entire body divided by BMI. | litres |
| 21 | **space2** | Space taken in by posture in relation to height | | Volume of 3D boundary around entire body divided by height. | litres |
| 22 | **space3** | Space taken in by posture in relation to weight | | Volume of 3D boundary around entire body divided by mass. | litres |
| **Kinematic Features** | | | | | |
| **Periodicity/Regularity of Walk** | | | | | |
| The modeling power is measure for the variance covered by the number of fourier components in the chosen model. Modeling an individual gait with FD requires the walk to be periodic and repetitive (in gait all limbs repeatedly perform the same movement). Non-periodic or irregular elements are reflected in a higher modeling error (1-MP) or a lower power of the FD. | | | | | |
| 25 | **Mpower** | Mean Power | | We were able to model the walk of each individual with two fourier components only. The variance carried by the residual term, not represented by the FD, was less than 1% in all walkers (err: min = 0.003, max. = 0.01).  The mean power represents the average variance covered by the two fourier components, compared to a model with more (in our case 4) fourier components (difference) averaged across 8 moves. |  |
| 26 | **SDpower** | Standard Deviation of Power | | Standard deviation of Mpower across 8 moves. Essentially: How much does the power of the Fourier modeling vary across the moves. |  |
| **Sway of Body Parts** | | | | | |
| Sway features quantify the movement of single body parts. Calculated from the limb amplitudes, they express the amount of movement of a certain body part in space. | | | | | |
| 27 | **WSL3** | Wrist Sway Left 3D | | Max. minus min. Amplitude of LWJC all directions (x,y,z). | mm |
| 28 | **WSR3** | Wrist Sway Right 3D | | Max minus min Amplitude of RWJC all directions (x,y,z). | mm |
| 29 | **DiffWSLR** | Non-directional Difference Wrist Sway Left Right | | Absolute values of difference of 3D left and right Wrist Sway. Difference close to 0: 3D Wrist Swing on both sides similar. Difference different to 0: one side moves more than the other. | mm |
| 30 | **RatioWSLR** | Non-directional Ratio Wrist Sway Left Right | | Ratio of 3D left and right Wrist Sway. Higher Value is divided by smaller value. Ratio close to 1: 3D Wrist Swing on both sides similar. Ratio bigger than 1: one side moves more than the other. |  |
| 31 | **AS3** | Arm Sway 3D | | Mean 3D Wrist Sway of Left and Right. WSL3 added to WSR3 and divided by 2. | mm |
| 32 | **WSL2** | Wrist Sway Left 2D | | Max. minus min. amplitude of left wrist in x and y direction. | mm |
| 32 | **WSR2** | Wrist Sway Right 2D | | Max. minus min. amplitude of right wrist in x and y direction. | mm |
| 34 | **AS2** | Arm Swing 2D | | Mean 2D wrist sway of the left and right side. | mm |
| 35 | **DiffWSLR2** | Non-directional Difference Wrist Sway Left Right 2D | | Absolute values of difference between 2D Wrist Sway of Left and right side. Smaller Value is substrated from larger value. | mm |
| 36 | **RatioWSLR2** | Non-directional Ratio Wrist Sway Left Right 2D | | Ratio of 2D Wrist Sway of Left and right side. Higher Value is divided by smaller value. | mm |
| 37 | **ASL1x** | Arm Swing Left 1D in x direction | | Max. minus min. amplitude of LWJC in x direction. | mm |
| 38 | **ASR1x** | Arm Swing Right 1D in x direction | | Max. minus min. Amplitude of RWJC in x direction. | mm |
| 39 | **AS1x** | Arms Swing 1D in x direction | | Mean wrist sway in x direction of left and right side. | mm |
| 40 | **DiffWSLR1** | Non-directional Difference Wrist Sway Left Right 1D in x direction | | Absolute values of difference between 1D wrist sway of left and right side in x direction. Smaller values are subtracted from bigger values. | mm |
| 41 | **RatioWSLR1** | Non-directional Ratio Wrist Sway Left Right 1D in x direction | | Ratio of 1D wrist sway of left and right side. Higher Value is divided by smaller value. So bigger ratio means more difference. |  |
| 42 | **ESL3** | Elbow Sway Left 3D | | Max. minus min. Amplitude of LEJC all directions (x,y,z). | mm |
| 43 | **ESR3** | Elbow Sway Right 3D | | Max. minus min. Amplitude of REJC all directions (x,y,z). | mm |
| 44 | **DiffESLR** | Non-directional Difference Elbow Sway Left Right | | Absolute values of difference of 3D left and right elbow sway (ESL3-ESR3). Smaller value is subtracted from bigger value. | mm |
| 45 | **RatioESLR** | Ratio Elbow Sway Left Right | | Ratio of 3D left and right Elbow Sway (ESL3/ESR3). Higher Value is divided by smaller value. |  |
| 46 | **ES3** | Elbow Sway 3D | | Mean 3D elbow sway of left and right side. | mm |
| 47 | **LBS** | Lateral Body Sway | | Mean amplitude of LSJC and RSJC in y direction. | mm |
| 48 | **SS3** | Shoulder Sway 3D | | Mean amplitude of LSJC and RSJC in all directions (x,y,z). | mm |
| 49 | **VBS** | Vertical Body Sway | | Max. minus min. amplitude of HEDO in z direction. | mm |
| 50 | **LHS** | Lateral Hips Sway | | Mean amplitude of LHJC and RHJC in y direction. | mm |
| 51 | **HS3** | Hip Sway 3D | | Mean Amplitude of LHJC and RHJC in all directions (x,y,z). | mm |
| 52 | **KSL3** | Knee Sway Left 3D | | Max. minus min. amplitude of LKJC all directions (x,y,z). | mm |
| 53 | **KSR3** | Knee Sway Right 3D | | Max. minus min. amplitude of RKJC all directions (x,y,z). | mm |
| 54 | **DiffKSLR** | Non-directional Difference Knee Sway Left Right | | Absolute values of difference of 3D left and right knee sway (KSL3-KSR3). Smaller value is subtracted from larger vaue. |  |
| 56 | **RatioKSLR** | Non-directional Ratio Knee Sway Left Right | | Ratio of 3D left and right Knee Sway (KSL3/KSR3). Higher value is divided by smaller value. | mm |
| 57 | **KS3** | Knee Sway 3D | | Mean amplitude of LKJC and RKJC in all directions (x,y,z). | mm |
| **Relation of Sway of Body Parts** | | | | | |
| Relational sway features look at the interplay of limb movement (interlimb coordination). Relational movement is operationalized as differences between and ratios of movement of single body parts – sometimes, when there is a left and a right side, as the relational movement of the averaged amplitudes of both sides. | | | | | |
| 58 | **RatioWEd** | Directional Ratio Wrist Elbow | | Ratio of mean wrist and elbow sway both sides (left/right) all directions (x,y,z). A ratio above 1: wrist moves more; below 1: elbow is used more. |  |
| 59 | **DiffWEd** | Directional Difference Wrist Elbow | | Difference mean wrist and elbow sway both sides (left/right) all directions (x,y,z). Positive Difference: wrist moves more, negative difference elbow moves more. | mm |
| 60 | **RatioLWEd** | Directional Ratio Left Wrist Elbow | | Ratio Left Wrist and Elbow Sway all directions (x,y,z) |  |
| 61 | **DiffLWEd** | Directional Difference Left Wrist Elbow | | Difference Left Wrist and Elbow Sway all directions (x,y,z) | mm |
| 62 | **RatioRWEd** | Directional Ratio Right Wrist Elbow | | Ratio Right Wrist and Elbow Sway all directions (x,y,z) |  |
| 63 | **DiffRWEd** | Directional Difference Right Wrist Elbow | | Difference Right Wrist and Elbow Sway all directions (x,y,z) | mm |
| 64 | **RatioLAd** | Directional Ratio Leg Arm | | Ratio of mean of amplitude of LAJC and RAJC in x direction and 2D Arm Sway. (2D and 1D Leg Sway are almost the same.) |  |
| 65 | **DiffLAd** | Directional Difference Leg Arm | | Difference of mean of amplitude of LAJC and RAJC in x direction and 2D Arm Sway. (2D and 1D Leg Sway are almost the same.) | mm |
| 66 | **RatioSHd** | Directional Ratio Shoulder Hip | | Lateral Body Sway (Shoulder) divided by Lateral Hip Sway (only in y direction). |  |
| 67 | **RatioSH3d** | Directional Ratio Shoulder Hip 3D | | Ratio of 3D shoulder and hip sway  (all directions). |  |
| 68 | **RatioSH** | Non-directional Ratio Shoulder Hip | | Ratio of Lateral Body and Hip Sway. Higher value divided by smaller value. Ratio above 1 indicates difference but no direction. |  |
| 69 | **RatioSH3** | Non-directional 3D Ratio Shoulder Hip | | Ratio of 3D shoulder sway and 3D hip sway. Larger value devided by smaller value. Ratio above 1 indicates difference but no direction. |  |
| 70 | **DiffSHd** | Directional Difference Shoulder Hip | | Lateral Body Sway (Shoulder) minus Lateral Hip Sway (only in y direction). | mm |
| 71 | **DiffSH3d** | Directional Difference Shoulder Hip 3D | | Difference of 3D shoulder and hip sway (all directions). | mm |
| 72 | **DiffSH** | Non-directional Difference Shoulder Hip | | Absolute values of difference between LBS and LHS. | mm |
| 73 | **DiffSH3** | Non-directional 3D Difference Shoulder Hip | | Absolute Values of difference between SS3 and HS3. | mm |
| **Activated/Utilized Range of Motion** | | | | | |
| The utilized range of motion (URM) is the physicality independent equivalent to subgroup the sway features. It does not only look at movement in space, but is operationalized as the variation (SD) of joint angles within one gait cycle of a subject. A larger person for example has a longer arm. The wrist or the elbow of a longer arm can swing more in space, which can result in a larger arm sway. Calculated with the time-series of joint angles, URM is not affected by the length of the arm. | | | | | |
| 74 | **URMLAx** | Utilized Range of Motion Left Arm in x direction | | Motion of upper left Arm x direction: Standard deviation of angle time series between difference in vertical line (z) (LSJC – LEJC), and LEJC in x direction.  Attention: URMLAx is physicality independent equivalent of Arm Sway (ASL1x)! It does not only look at movement in space, but is operationalized as the variation (SD) of joint angles within one gait cycle of a subject. | degree |
| 75 | **URMRAx** | Utilized Range of Motion Right Arm in x direction | | Motion of upper right arm x direction: Standard deviation of angle time series between difference in vertical line (z) (RSJC – REJC), and REJC in x direction | degree |
| 76 | **URMLAy** | Utilized Range of Motion Left Arm in y direction | | Motion of upper left Arm y direction: Standard deviation of time series of angle between difference in vertical line (z) (LSJC and LEJC), and LEJC in y direction. | degree |
| 77 | **URMRAy** | Utilized Range of Motion Right Arm in x direction | | Motion of upper right Arm y direction: Standard deviation of time series of angle between difference in vertical line (z) (RSJC - REJC), and REJC in y direction. | degree |
| 78 | **URMLEx** | Utilized Range of Motion Left Elbow in x direction | | Motion of lower left Arm x direction: Standard deviation of time series of angle between difference in vertical line (z) (LEJC – LWJC), and LWJC in x direction. | degree |
| 79 | **URMREx** | Utilized Range of Motion Right Elbow in x direction | | Motion of lower right Arm x direction: Standard deviation of time series of angle between difference in vertical line (z) (LEJC – LWJC), and LWJC in x direction. | degree |
| 80 | **URMLEy** | Utilized Range of Motion Left Elbow in y direction | | Motion of lower left Arm y direction: Standard deviation of time series of angle between difference in vertical line (z) (LEJC - LWJC), and LWJC in y direction. | degree |
| 81 | **URMREy** | Utilized Range of Motion Right Elbow in y direction | | Motion of lower right Arm y direction: Standard deviation of time series of angle between difference in vertical line (z) (LEJC and LWJC), and LWJC in y direction. | degree |
| 82 | **URMLE3** | Utilized Range of Motion Left Elbow 3D | | Standard deviation of time series of 3D angle between LSJC, LEJC, LWJC. | degree |
| 83 | **URMRE3** | Utilized Range of Motion Right Elbow 3D | | Standard deviation of time series of 3D angle between RSJC, REJC, RWJC. | degree |
| 84 | **URMTR2** | Utilized Range of Motion Thorax 2D | | Standard Deviation of Time Series of 2D (x and y) angle between vertical line and TRXO | degree |
| 85 | **URMTRy** | Utilized Range of Motion Thorax in y direction | | Standard deviation of time series of angle between vertical line and TRXO in y direction | degree |
| 86 | **URMLS2** | Utilized Range of Motion Left Shoulder 2D | | Standard deviation of 2D angles of LSJC (x, y) to BMLPEL as origin. | degree |
| 87 | **URMRS2** | Utilized Range of Motion Right Shoulder 2D | | Standard deviation of 2D angles of LSJC (x, y) to BMLPEL as origin | degree |
| 88 | **URMLSy** | Utilized Range of Motion Left Shoulder in y direction | | Standard deviation of Times series of angles of LSJC in y direction to BMLPEL as origin.  Attention: URMLSy is not the same than Shoulder Sway (SS3)!  Because BMLPEL also moves in y direction over time, URMLS is the movement of the shoulder only (independent of the movement of the whole body). Shoulder sway instead is just the amplitude of the shoulder in space, which depends on the sway of the entire body. | degree |
| 89 | **URMRSy** | Utilized Range of Motion Right Shoulder in y direction | | Standard deviation of times series of angles of RSJC in y direction to BMLPEL as origin. | degree |
| 90 | **URMLSx** | Utilized Range of Motion Left Shoulder in x direction | | Standard deviation of times series of angles of LSJC in x direction to BMLPEL as origin. | degree |
| 91 | **URMRSx** | Utilized Range of Motion Right Shoulder in x direction | | Standard deviation of times series of angles of RSJC in x direction to BMLPEL as origin. | degree |
| 92 | **URMLSz** | Utilized Range of Motion Left Shoulder in z direction | | Standard deviation of times series of angles of LSJC in z direction to BMLPEL as origin | degree |
| 93 | **URMRSz** | Utilized Range of Motion Right Shoulder in z direction | | Standard deviation of times series of angles of RSJC in z direction to BMLPEL as origin. | degree |
| 94 | **URMLH2** | Utilized Range of Motion Left Hip 2D | | Standard deviation of time series of 2D angle (2D distance in space) between vertical line (BMLPEL as origin) and LHJC (x and y direction). | degree |
| 95 | **URMRH2** | Utilized Range of Motion Right Hip 2D | | Standard deviation of time series of 2D angle between vertical line (BMLPEL as origin) and RHJC (x and y direction). | degree |
| 96 | **URMLHy** | Utilized Range of Motion Left Hip in y direction | | Standard deviation of times series of angle of LHJC in y direction to BMLPEL as origin. | degree |
| 97 | **URMRHy** | Utilized Range of Motion Right Hip in y direction | | Standard deviation of times series of angle of RHJC in y direction to BMLPEL as origin. | degree |
| 98 | **URMLHx** | Utilized Range of Motion Left Hip in x direction | | Standard deviation of times series of angle of LHJC in x direction to BMLPEL as origin. | degree |
| 99 | **URMRHx** | Utilized Range of Motion Right Hip in x direction | | Standard deviation of times series of angle of RHJC in x direction to BMLPEL as origin. | degree |
| 100 | **URMLHz** | Utilized Range of Motion Left Hip in z direction | | Standard deviation of times series of angle of LHJC in z direction to BMLPEL as origin. | degree |
| 101 | **URMRHz** | Utilized Range of Motion Right Hip in z direction | | Standard deviation of times series of angle of RHJC in z direction to BMLPEL as origin. | degree |
| 102 | **URMLK3** | Utilized Range of Motion Left Knee 3D | | Standard Deviation of time Series of 3D angle between LHJC, LKJC, LAJC for one gait cycle. | degree |
| 103 | **URMRK3** | Utilized Range of Motion Right Knee 3D | | Standard Deviation of time Series of 3D angle between RHJC, RKJC, RAJC for one gait cycle. | degree |
| **Relational Utilized Range of Motion** | | | | | |
| The relational URM relates changes in angle movement of different joints as well as into different directions to each other. It is the physicality-independent and more detailed equivalent to the relational sway features. | | | | | |
| **Relation of movement in anterior-posterior and lateral direction** | | | | | |
| 104 | **ratioURMLA** | Ratio Utilized Range of Motion Left Arm | | Ratio of URM of left upper arm in x direction to URM of left upper arm in y direction.  A Ratio bigger than 1: more movement in x direction; smaller than 1: more movement in y direction. A ratio close to 1: similar/same amount of movement. |  |
| 105 | **diffURMLA** | Difference Utilized Range of Motion Left Arm | | Difference of URM of left upper arm in x and URM of left upper arm in y direction.  A Difference bigger than 0: more movement in x direction; smaller than 0: more movement in y direction. A ratio close to 0: similar/same amount of movement. |  |
| 106 | **ratioURMRA** | Ratio Utilized Range of Motion Right Arm | | Ratio of URM of right upper arm in x direction to URM of right upper arm in y direction. |  |
| 107 | **diffURMRA** | Difference Utilized Range of Motion Right Arm | | Difference of URM of right upper arm in x and URM of right upper arm in y direction. |  |
| 108 | **ratioURMLE** | Ratio Utilized Range of Motion Left Elbow | | Ratio of URM of left lower arm in x direction to URM of left lower arm in y direction. |  |
| 109 | **diffURMLE** | Difference Utilized Range of Motion Left Elbow | | Difference of URM of left lower arm in x and URM of left lower arm in y direction. |  |
| 110 | **ratioURMRE** | Ratio Utilized Range of Motion Right Elbow | | Ratio of URM of right lower arm in x direction to URM of right lower arm in y direction. |  |
| 111 | **diffURMRE** | Difference Utilized Range of Motion Right Elbow | | Difference of URM of right lower arm in x direction and URM of right lower arm in y direction. |  |
| 112 | **ratioURMLS** | Ratio Utilized Range of Motion Left Shoulder | | Ratio of URM of Left shoulder in x direction to URM of left shoulder in y direction. |  |
| 113 | **diffURMLS** | Difference Utilized Range of Motion Left Shoulder | | Difference of URM of left shoulder in x direction and URM of left shoulder in y direction. |  |
| 114 | **ratioURMRS** | Ratio Utilized Range of Motion Right Shoulder | | Ratio of URM of Right shoulder in x and y direction |  |
| 115 | **diffURMRS** | Difference Utilized Range of Motion Right Shoulder | | Difference of URM of right shoulder between x and y direction. |  |
| 116 | **ratioURMLH** | Ratio Utilized Range of Motion Left Hip | | Ratio of URM of left hip in x and y direction. |  |
| 117 | **diffURMLH** | Difference Utilized Range of Motion Left Hip | | Difference of URM of right hip between x and y direction. |  |
| 118 | **ratioURMRH** | Ratio Utilized Range of Motion Right Hip | | Ratio of URM of right hip in x and y direction. |  |
| 119 | **diffURMRH** | Difference Utilized Range of Motion Right Hip | | Difference of URM of right hip between x and y direction. |  |
| **Relation of movement of different joints** | | | | | |
| 120 | **ratioLELAx** | Ratio left Elbow left Arm in x direction | | Ratio of URM of left upper arm (arm) and left lower arm (elbow) in x direction. Ratio close to 1: stiffness of arm, both parts move similar much; A ratio smaller than 1: upper arm moves more; A Ratio bigger than 1: lower arm moves more. |  |
| 121 | **diffLELAx** | Difference left Elbow left Arm in x direction | | Difference of URM of left upper arm (arm) and left lower arm (elbow) in x direction. A difference close to 0: stiffness of arm, both parts move similar much; Diff below 0: upper arm moves more; Difference above 0: lower arm moves more. |  |
| 122 | **ratioLELAy** | Ratio left Elbow left Arm in y direction | | Ratio of URM of left upper arm (arm) and left lower arm (elbow) in y direction. Ratio close to 1: stiffness of arm, both parts move similar much; Ratio below 1: upper arm moves more; Ratio above 1: lower arm moves more. |  |
| 123 | **diffLELAy** | Difference left Elbow left Arm in y direction | | Difference of URM of left upper arm (arm) and left lower arm (elbow) in x direction. Differene close to 0: stiffness of arm, both parts move similar much; difference below 0: upper arm moves more; difference above 0: lower arm moves more. |  |
| 124 | **ratioRERAx** | Ratio right Elbow right Arm in x direction | | Ratio of URM of right upper arm (arm) and right lower arm (elbow) in x direction. Ratio close to 1: stiffness of arm; both parts move similar much; Ratio below 1: upper arm moves more; Ratio above 1: lower arm moves more. |  |
| 125 | **diffRERAx** | Difference right Elbow right Arm in x direction | | Difference of URM of right upper arm (arm) and right lower arm (elbow) in x direction. Difference close to 0: stiffness of arm, both parts move similar much; Diff below 0: upper arm moves more; Difference above 0: lower arm moves more. |  |
| 126 | **ratioRERAy** | Ratio right Elbow right Arm in y direction | | Ratio of URM of right upper arm (arm) and right lower arm (elbow) in y direction. Ratio close to 1: stiffness of arm, both parts move similar much. Ratio below 1: upper arm moves more; Ratio above 1: lower arm moves more. |  |
| 127 | **diffRERAy** | Difference right Elbow right Arm in y direction | | Difference of URM of right upper arm (arm) and right lower arm (elbow) in y direction. Difference close to 0: stiffness of arm, both parts move similar much; Difference below 0: upper arm moves more. Difference above 0: lower arm moves more. |  |
| 128 | **ratioLSH2** | Ratio Left Shoulder Hip 2D | | Ratio of URM of left shoulder and hip in 2D (x and y). |  |
| 129 | **diffLSH2** | Difference Left Shoulder Hip 2D | | Difference of URM between left shoulder and hip in 2D (x and y). |  |
| 130 | **ratioRSH2** | Ratio Right Shoulder Hip 2D | | Ratio of URM of right shoulder and hip in 2D (x and y) |  |
| 131 | **diffRSH2** | Difference Right Shoulder Hip 2D | | Difference of URM between right shoulder and hip in 2D (x and y). |  |
| 132 | **ratioLSHy** | Ratio Left Shoulder Hip in y direction | | Ratio close to 1: stiffness of upper body, shoulders and hips move similar much. Ratio below 1: hip moves more; Ratio above 1: shoulder moves more. |  |
| 133 | **diffLSHy** | Difference Left Shoulder Hip in y direction | | Difference close to 0: stiffness of upper body, shoulders and hips move similar much.  Difference below 0: Hip moves more; Difference above 0: shoulder moves more. |  |
| 134 | **ratioRSHy** | Ratio Right Shoulder Hip in y direction | | Ratio close to 1: stiffness of upper body, shoulders and hips move similar much. Ratio below 1: hip moves more; Ratio above 1: shoulder moves more. |  |
| 135 | **diffRSHy** | Difference Right Shoulder Hip in y direction | | Difference close to 0: stiffness of upper body, shoulders and hips move similar much.  Difference below 0: Hip moves more; Difference above 0: shoulder moves more. |  |
| 136 | **ratioLSHx** | Ratio Left Shoulder Hip in x direction | | Ratio close to 1: stiffness of upper body, shoulders and hips move similar much. Ratio below 1: hip moves more; Ratio above 1: shoulder moves more. |  |
| 137 | **diffLSHx** | Difference Left Shoulder Hip in x direction | | Difference close to 0: stiffness of upper body, shoulders and hips move similar much.  Difference below 0: Hip moves more; Difference above 0: shoulder moves more. |  |
| 138 | **ratioRSHx** | Ratio Right Shoulder Hip in x direction | | Ratio close to 1: stiffness of upper body, shoulders and hips move similar much. Ratio below 1: hip moves more; Ratio above 1: shoulder moves more. |  |
| 139 | **diffRSHx** | Difference Right Shoulder Hip in x direction | | Difference close to 0: stiffness of upper body, shoulders and hips move similar much.  Difference below 0: Hip moves more; Difference above 0: shoulder moves more. |  |
| 140 | **ratioLSHz** | Ratio Left Shoulder Hip in z direction | | Ratio close to 1: stiffness of upper body, shoulders and hips move similar much. Ratio below 1: hip moves more; Ratio above 1: shoulder moves more. |  |
| 141 | **diffLSHz** | Difference Left Shoulder Hip in z direction | | Difference close to 0: stiffness of upper body, shoulders and hips move similar much.  Difference below 0: Hip moves more; Difference above 0: shoulder moves more. |  |
| 142 | **ratioRSHz** | Ratio Right Shoulder Hip in z direction | | Ratio close to 1: stiffness of upper body, shoulders and hips move similar much. Ratio below 1: hip moves more; Ratio above 1: shoulder moves more. |  |
| 143 | **diffRSHz** | Difference Right Shoulder Hip in z direction | | Difference close to 0: stiffness of upper body, shoulders and hips move similar much.  Difference below 0: Hip moves more; Difference above 0: shoulder moves more. |  |
| 144 | **FUB** | Flexibility of Upper Body | | Relation of shoulder and hip movement 3D: Standard deviation of Angle (of one gait cycle) of dot product of 3D vectors of shoulder and hip joints. Higher SD, more variation, more flexibility. | degree |
| 145 | **FUBf** | Flexibility of Upper Body frontal | | Relation of Shoulder and Hip movement 2D in frontal plane: Standard deviation of Angle (of one gait cycle) of dot product of 2D (y and z) vectors of shoulder and hip joints. Higher SD, more variation, more flexibility. | degree |
| 146 | **FUBs** | Flexibility of Upper Body sagittal | | Relation of Shoulder and Hip movement 2D in sagittal plane: Standard deviation of Angle (of one gait cycle) of dot product of 2D (x and z) vectors of shoulder and hip joints. Higher SD, more variation, more flexibility. | degree |
| 147 | **FUBt** | Flexibility of Upper Body transversal | | Relation of Shoulder and Hip movement 2D in transversal plane: Standard deviation of Angle (of one gait cycle) of dot product of 2D (x and y) vectors of shoulder and hip joints. Higher SD, more variation, more flexibility. | degree |
| **Variation of Center of Mass (COM)** | | | | | |
| The variation (SD) of the COM describes changes in the movement of the centre of the body in different directions (anterior-posterior, lateral and vertical). | | | | | |
| 144 | **VCoMx** | Variation Center of Mass x | | Standard deviation of time series of position of full body Center of Mass in x direction. Full body Center of Mass is aggregated from CoM of 10 body segments. Little variation in x: no "Stockung"/staccato in Gait. |  |
| 145 | **VCoMy** | Variation Center of Mass y | | Standard deviation of time series of position of full body Center of Mass in y direction. Full body Center of Mass is aggregated from CoM of 10 Body segments. Little variation in z and y: smooth gait/balanced gait. |  |
| 146 | **VCoMz** | Variation Center of Mass z | | Standard deviation of time series of position of full body Center of Mass in z direction. Full body Center of Mass is aggregated from CoM of 10 Body segments. Little variation in z and y: smooth gait/balanced gait. |  |
| **Velocities of Body Parts** | | | | | |
| Subgroup velocities of body parts looks at the speed single limbs are used with. | | | | | |
| 147 | **vWSL2** | Velocity Wrist Sway Left 2D | | Two dimensional left wrist sway multiplied by cadence.  (see above for definition of WSL2) | mm/s  (multiple of cycle) |
| 148 | **vWSR2** | Velocity Wrist Sway Right 2D | | Two dimensional right wrist sway multiplied by cadence.  (See above for definition of WSR2) | mm/s |
| 149 | **vWSL3** | Velocity Wrist Sway Left 3D | | Three dimensional left wrist sway multiplied by cadence.  (see above for definition of WSL3) | mm/s |
| 150 | **vWSR3** | Velocity Wrist Sway Right 3D | | Three dimensional right wrist sway multiplied by cadence.  (see above for Definition of WSR3) | mm/s |
| 151 | **vESL3** | Velocity Elbow Sway Left 3D | | Three dimensional left elbow sway multiplied by cadence.  (see above for definition of ESL3) | mm/s |
| 152 | **vESR3** | Velocity Elbow Sway Right 3D | | Three dimensional right elbow Sway multiplied by cadence.  (see above for Definition of ESR3) | mm/s |
| 153 | **vKSL3** | Velocity Knee Sway Left 3D | | Three dimensional left knee Sway multiplied by cadence.  (see above for definition of KSL3) | mm/s |
| 154 | **vKSR3** | Velocity Knee Sway Right 3D | | Three dimensional right knee Sway multiplied by cadence.  (see above for Definition of ESR3) | mm/s |
| **Relational Velocities of Body Parts** | | | | | |
| Subgroup relational velocities of body parts compares the speed of different limb movement. | | | | | |
| 155 | **ratiovLKE3** | Ratio Velocity Left Knee Elbow 3D | | Ratio of velocity of three dimensional left elbow and knee Sway. |  |
| 156 | **diffvLKE3** | Difference Velocity Left Knee Elbow 3D | | Difference of velocity of three dimensional left elbow and knee sway. |  |
| 157 | **ratiovRKE3** | Ratio Velocity Right Knee Elbow 3D | | Ratio of velocity of three dimensional right elbow and knee sway. |  |
| 158 | **diffvRKE3** | Difference Right Knee Elbow 3D | | difference of velocity of three dimensional right elbow and knee sway. |  |

**Note**. *The 15 “virtual markers” or joint centers are mentioned with their abbreviations: HEDO = center of the head, TRXO = center of the sternum, LSJC = left shoulder joint center, RSJC = right shoulder joint center, LEJC = left elbow joint center, REJC = right elbow joint center, LWJC = left wrist joint center, RWJC = right wrist joint center, BMLPEL = center of the pelvis, LHJC = left hip joint center, RHJC = right hip joint center, LKJC = left knee joint center, RKJC = right knee joint center, LAJC = left ankle joint center, RAJC = right ankle joint center. Axes labels: x = anterior-posterior (AP), y = lateral, z = vertical. One stride consists of two steps. Those readers, who find step related variables more intuitive than stride related variables, can work with the variables highlighted in grey.*

**Table 2. Two-sided t-tests for all extracted movement features**

| **Feature** | ***M*** | | | **95% CI** | | ***t*_(38)_** | ***p*** | **Cohen’s d** |
| --- | --- | --- | --- | --- | --- | --- | --- | --- |
|  | **Patient**  **(N=20)** | | **Control**  **(N=20)** | **LL** | **UL** |  |  |  |
| **a) Basic Features** | | | | | | |  |  |
| **MV** | **1.084** | | **1.209** | **-0.223** | **-0.027** | **-2.571** | **0.014*** | **-0.813** |
| SDV | 0.037 | | 0.027 | -0.006 | 0.025 | 1.232 | 0.225 | 0.390 |
| CT | 1.129 | | 1.108 | -0.039 | 0.081 | 0.706 | 0.485 | 0.223 |
| ST | 0.564 | | 0.553 | -0.196 | 0.041 | 0.706 | 0.485 | 0.223 |
| CA | 0.893 | | 0.907 | -0.059 | 0.030 | -0.656 | 0.516 | -0.207 |
| SF | 1.786 | | 1.815 | -0.118 | 0.060 | -0.656 | 0.516 | -0.207 |
| SDCA | 0.022 | | 0.015 | -0.000 | 0.013 | 1.909 | 0.064 | 0.604 |
| SDSF | 0.044 | | 0.031 | -0.000 | 0.027 | 1.909 | 0.064 | 0.604 |
| **MSTRL** | **1.215** | | **1.330** | **-0.202** | **-0.029** | **-2.696** | **0.010*** | **-0.853** |
| **MSL** | **0.607** | | **0.665** | **-0.101** | **-0.014** | **-2.696** | **0.010*** | **-0.853** |
| SDSTRL | 0.035 | | 0.028 | -0.007 | 0.020 | 0.961 | 0.343 | 0.304 |
| SDSL | 0.017 | | 0.014 | -0.003 | 0.010 | 0.961 | 0.343 | 0.304 |
| Regularity of Walk | | | | | | | | |
| **MPower** | | **0.993** | **0.995** | **-0.002** | **-0.001** | **-3.971** | **0.000***** | **-1.256** |
| **SDPower** | | **0.0008** | **0.0005** | **0.0001** | **0.0003** | **2.899** | **0.006**** | **0.917** |
| **b) Postural Features** | | | | | | | | |
| **alphaHEAD** | **12.867** | | **5.301** | **3.854** | **11.274** | **4.127** | **0.000**** | **1.309** |
| alphaBMLPEL | 4.965 | | 4.938 | -2.148 | 2.201 | 0.025 | 0.980 | 0.008 |
| alphaTRX | 185.25 | | 183.411 | -5.135 | 8.818 | 0.534 | 0.596 | 0.169 |
| alphaLA | 12.636 | | 10.709 | -2.043 | 5.898 | 0.982 | 0.332 | 0.311 |
| alphaRA | 14.753 | | 13.383 | -2.394 | 5.134 | 0.737 | 0.466 | 0.233 |
| alphaLK | 136.197 | | 138.956 | -5.803 | 0.284 | -1.836 | 0.074 | -0.581 |
| alphaRK | 137.064 | | 138.788 | -4.920 | 1.472 | -1.092 | 0.282 | -0.345 |
| space | 2.022 | | 2.056 | -0.289 | 0.222 | -0.266 | 0.792 | -0.084 |
| **c) Sway of Body Parts** | | | | | | | | |
| **WSL3** | | **261.246** | **418.665** | **-240.747** | **-74.091** | **-3.824** | **0.000***** | **-1.209** |
| **WSR3** | | **217.250** | **384.860** | **-240.966** | **-73.872** | **-4.652** | **0.000***** | **-1.471** |
| **AS3** | | **239.248** | **401.762** | **-234.735** | **-90.294** | **-4.555** | **0.000***** | **-1.441** |
| **WSL2** | | **247.086** | **396.892** | **-230.792** | **-68.818** | **-3.745** | **0.001**** | **-1.184** |
| **WSR2** | | **203.823** | **366.547** | **-234.778** | **-90.670** | **-4.572** | **0.000***** | **-1.446** |
| **AS2** | | **225.55** | **381.719** | **-226.952** | **-85.577** | **-4.475** | **0.000***** | **-1.415** |
| **ASL1x** | | **240.210** | **392.976** | **-232.807** | **-72.724** | **-3.864** | **0.000***** | **-1.222** |
| **ASR1x** | | **169.308** | **363.490** | **-239.992** | **-94.371** | **-4.648** | **0.000***** | **-1.470** |
| **AS1x** | | **218.259** | **378.233** | **-230.592** | **-89.355** | **-4.586** | **0.000***** | **-1.450** |
| **ESL3** | | **141.424** | **202.882** | **-95.531** | **-27.385** | **-3.651** | **0.001**** | **-1.155** |
| **ESR3** | | **126.004** | **185.913** | **-88.892** | **-30.926** | **-4.185** | **0.000***** | **-1.323** |
| **ES3** | | **133.714** | **194.397** | **-90.415** | **-30.952** | **-4.132** | **0.000***** | **-1.307** |
| **LBS** | | **41.841** | **32.612** | **3.143** | **15.317** | **3.070** | **0.004**** | **0.971** |
| VBS | | 36.630 | 37.939 | -7.626 | 5.007 | -0.420 | 0.677 | -0.133 |
| SS3 | | 71.259 | 69.335 | -6.405 | 10.253 | 0.468 | 0.643 | 0.148 |
| LHS | | 26.404 | 31.604 | -11.337 | 0.937 | -1.715 | 0.094 | -0.542 |
| HS3 | | 61.308 | 67.400 | -12.700 | 0.516 | -1.866 | 0.070 | -0.590 |
| **KSL3** | | **299.171** | **323.829** | **-48.540** | **-0.776** | **-2.090** | **0.043*** | **-0.661** |
| **KSR3** | | **289.709** | **321.029** | **-55.098** | **-7.541** | **-2.666** | **0.011**** | **-0.843** |
| **KS3** | | **294.440** | **322.429** | **-51.227** | **-4.751** | **-2.438** | **0.020*** | **-0.771** |
| **d) Relational Sway of Body Parts** | | | | | | | | |
| Relation of Body Sides | | | | | | | | |
| DiffWSLR | | 72.356 | 78.095 | -49.129 | 37.651 | -0.268 | 0.790 | -0.085 |
| RatioWSLR | | 1.455 | 1.255 | -0.066 | .0466 | 1.525 | 0.136 | 0.482 |
| DiffWSLR2 | | 73.032 | 74.974 | -43.821 | 39.936 | -0.094 | 0.926 | -0.030 |
| RatioWSLR2 | | 1.539 | 1.256 | -0.019 | 0.584 | 1.898 | 0.065 | 0.600 |
| DiffWSLR1 | | 77.228 | 73.553 | -36.552 | 43.902 | 0.185 | 0.854 | 0.058 |
| **RatioWSLR1** | | **1.753** | **1.255** | **0.0176** | **0.979** | **2.098** | **0.043*** | **0.664** |
| DiffESLR | | 25.161 | 31.675 | -21.566 | 8.539 | -0.876 | 0.387 | -0.277 |
| RatioESLR | | 1.230 | 1.191 | -0.0830 | 0.162 | 0.650 | 0.519 | 0.206 |
| DiffKSLR | | 15.936 | 15.120 | -4.520 | 6.153 | 0.310 | 0.759 | 0.098 |
| RatioKSLR | | 1.0573 | 1.050 | -0.0124 | 0.0277 | 0.777 | 0.442 | 0.246 |
| Relation of Body Parts | | | | | | | | |
| **RatioLWEd** | | **1.781** | **2.063** | **-0.502** | **-0.062** | **-2.595** | **0.013*** | **-0.821** |
| **DiffLWEd** | | **119.822** | **215.783** | **-149.110** | **-42.811** | **-3.655** | **0.001**** | **-1.156** |
| **RatioRWEd** | | **1.663** | **2.054** | **-0.625** | **-0.156** | **-3.375** | **0.002**** | **-1.067** |
| **DiffRWEd** | | **91.246** | **198.947** | **-155.360** | **-60.041** | **-4.575** | **0.000***** | **-1.447** |
| **RatioWEd** | | **1.738** | **2.062** | **-0.526** | **-0.123** | **-3.260** | **0.002**** | **-1.031** |
| **DiffWEd** | | **105.534** | **207.365** | **-147.872** | **-55.789** | **-4.477** | **0.000***** | **-1.416** |
| **RatioLAd** | | **3.479** | **1.929** | **0.379** | **2.721** | **2.679** | **0.011*** | **0.847** |
| **DiffLAd** | | **394.516** | **282.596** | **39.225** | **184.616** | **3.117** | **0.003**** | **0.986** |
| RatioSH | | 1.895 | 1.693 | -0.426 | 0.831 | 0.652 | 0.518 | 0.206 |
| DiffSH | | 16.206 | 12.796 | -3.654 | 10.472 | 0.977 | 0.335 | 0.309 |
| RatioSH3 | | 1.239 | 1.206 | -0.0689 | 0.136 | 0.661 | 0.512 | 0.209 |
| DiffSH3 | | 13.963 | 12.205 | -4.190 | 7.706 | 0.598 | 0.553 | 0.189 |
| **RatioSHd** | | **1.878** | **1.172** | **0.170** | **1.241** | **2.666** | **0.011*** | **0.843** |
| **DiffSHd** | | **15.437** | **1.007** | **4.931** | **23.929** | **3.075** | **0.004**** | **0.972** |
| RatioSH3d | | 1.180 | 1.049 | -0.014 | 0.276 | 1.825 | 0.076 | 0.577 |
| DiffSH3d | | 9.951 | 1.935 | -1.333 | 17.365 | 1.736 | 0.091 | 0.549 |
| **e) Utilized Range of Motion** | | | | | | | | |
| **URMLAx** | | **9.753** | **15.027** | **-8.276** | **-2.271** | **-3.556** | **0.001**** | **-1.124** |
| **URMRAx** | | **8.641** | **13.647** | **-7.538** | **-2.473** | **-4.001** | **0.000***** | **-1.265** |
| URMLAy | | 2.347 | 2.561 | -0.7816 | 0.355 | -0.760 | 0.452 | -0.240 |
| **URMRAy** | | **1.529** | **2.043** | **-0.880** | **-0.147** | **-2.835** | **0.007**** | **-0.896** |
| **URMLEx** | | **19.342** | **30.572** | **-17.228** | **-5.231** | **-3.790** | **0.001**** | **-1.199** |
| **URMREx** | | **15.601** | **28.653** | **-18.722** | **-7.381** | **-4.660** | **0.000***** | **-1.474** |
| **URMLEy** | | **3.617** | **6.254** | **-4.310** | **-0.964** | **-3.191** | **0.003**** | **-1.009** |
| **URMREy** | | **2.154** | **5.072** | **-4.103** | **-1.732** | **-4.980** | **0.000***** | **-1.575** |
| **URMLE3** | | **3.988** | **6.485** | **-4.339** | **-0.654** | **-2.743** | **0.009**** | **-0.867** |
| **URMRE3** | | **3.618** | **6.956** | **-5.251** | **-1.425** | **-3.533** | **0.001**** | **-1.117** |
| URMTR2 | | 0.702 | 0.615 | -0.114 | 0.289 | 0.876 | 0.386 | 0.277 |
| **URMTRy** | | **1.964** | **1.559** | **0.128** | **0.681** | **2.965** | **0.005**** | **0.938** |
| URMLS2 | | 0.614 | 0.747 | -0.315 | 0.481 | -1.488 | 0.145 | -0.470 |
| URMRS2 | | 0.593 | 0.712 | -0.295 | 0.566 | -1.373 | 0.178 | -0.434 |
| **URMLSy** | | **1.972** | **1.656** | **0.001** | **0.631** | **2.032** | **0.049*** | **0.643** |
| **URMRSy** | | **1.958** | **1.590** | **.053** | **.682** | **2.369** | **.023*** | **.749** |
| **URMLSx** | | **1.308** | **1.757** | **-.669** | **-.229** | **-4.127** | **.000**** | **-1.305** |
| URMRSx | | 1.695 | 1.926 | -.602 | .140 | -1.261 | .215 | -.399 |
| URMLSz | | .407 | .320 | -.009 | .183 | 1.826 | .076 | .577 |
| **URMRSz** | | **.394** | **.298** | **.002** | **.189** | **2.057** | **.047*** | **.651** |
| URMLH2 | | 1.298 | 1.503 | -.573 | .163 | -1.127 | .267 | -.356 |
| **URMRH2** | | **1.197** | **1.584** | **-.748** | **-.026** | **-2.168** | **.037*** | **-.686** |
| URMLHy | | 3.219 | 4.079 | -1.754 | .034 | -1.948 | .059 | -.616 |
| URMRHy | | 3.127 | 3.919 | -1.681 | .098 | -1.802 | .080 | -.570 |
| URMLHx | | 3.764 | 4.783 | -2.137 | .099 | -1.846 | .073 | -.584 |
| **URMRHx** | | **3.506** | **4.487** | **-1.864** | **-.097** | **-2.247** | **.031*** | **-.711** |
| **URMLHz** | | **.351** | **.437** | **-.171** | **-.002** | **-2.067** | **.046*** | **-.654** |
| URMRHz | | .365 | .418 | -.142 | .036 | -1.212 | .233 | -.383 |
| URMLK3 | | 17.135 | 17.349 | -1.333 | .905 | -.387 | .701 | -.122 |
| URMRK3 | | 16.922 | 17.782 | -1.840 | .119 | -1.778 | .083 | -.562 |
| **f) Relational Utilized Range of Motion** | | | | | | | | |
| Relation of movement in x (walking direction) and y (sideways) direction | | | | | | | | |
| **ratioURMLA** | | **4.518** | **6.212** | **-3.083** | **-.303** | **-2.466** | **.018*** | **-.780** |
| **diffURMLA** | | **7.406** | **12.466** | **-7.820** | **-2.300** | **-3.712** | **.001**** | **-1.174** |
| ratioURMRA | | 6.068 | 7.364 | -3.293 | .702 | -1.313 | .197 | -.415 |
| **diffURMRA** | | **7.112** | **11.604** | **-7.0166** | **-1.967** | **-3.602** | **.001**** | **-1.139** |
| ratioURMLE | | 5.993 | 5.488 | -1.075 | 2.085 | .647 | .521 | .205 |
| **diffURMLE** | | **15.725** | **24.318** | **-13.393** | **-3.792** | **-3.624** | **.001**** | **-1.146** |
| ratioURMRE | | 7.807 | 6.104 | -.268 | 3.675 | 1.749 | .088 | .553 |
| diffURMRE | | 6.487 | 8.575 | -4.251 | .075 | -1.954 | .058 | -.618 |
| **ratioURMLS** | | **.685** | **1.152** | **-.668** | **-.266** | **-4.704** | **.000***** | **-1.488** |
| **diffURMLS** | | **-.664** | **.101** | **-1.090** | **-.441** | **-4.776** | **.000***** | **-1.510** |
| **ratioURMRS** | | **.867** | **1.330** | **-.729** | **-.197** | **-3.520** | **.001**** | **-1.113** |
| **diffURMRS** | | **-.262** | **.337** | **-.966** | **-.231** | **-3.302** | **.002**** | **-1.044** |
| ratioURMLH | | 2.667 | 1.291 | -1.537 | 4.287 | .956 | .345 | .302 |
| diffURMLH | | .545 | .704 | -1.561 | 1.242 | -.230 | .819 | -.073 |
| ratioURMRH | | 1.915 | 1.454 | -1.207 | 2.128 | .559 | .579 | .177 |
| diffURMRH | | .379 | .568 | -1.386 | 1.008 | -.320 | .751 | -.101 |
| Relation of movement of different joints | | | | | | | | |
| ratioLELAx | | 1.934 | 2.103 | -.424 | .086 | -1.341 | .188 | -.424 |
| **diffLELAx** | | **9.589** | **15.545** | **-9.441** | **-2.471** | **-3.459** | **.001**** | **-1.094** |
| **ratioLELAy** | | **1.502** | **2.468** | **-1.459** | **-.472** | **-3.964** | **.000***** | **-1.253** |
| **diffLELAy** | | **1.270** | **3.693** | **-3.719** | **-1.128** | **-3.787** | **.001**** | **-1.198** |
| **ratioRERAx** | | **1.717** | **2.125** | **-.672** | **-.145** | **-3.136** | **.003**** | **-.992** |
| **diffRERAx** | | **6.960** | **15.006** | **-11.674** | **-4.418** | **-4.490** | **.000***** | **-1.420** |
| **ratioRERAy** | | **1.459** | **2.625** | **-1.809** | **-.523** | **-3.673** | **.001**** | **-1.161** |
| **diffRERAy** | | **.625** | **3.029** | **-3.479** | **-1.329** | **-4.528** | **.000***** | **-1.432** |
| ratioLSH2 | | .609 | .547 | -.175 | .299 | .531 | .598 | .168 |
| diffLSH2 | | -.684 | -.756 | -.325 | .468 | .365 | .717 | .116 |
| ratioRSH2 | | .850 | .517 | -.293 | .961 | 1.078 | .288 | .341 |
| diffRSH2 | | -.605 | -.872 | -.133 | .668 | 1.353 | .184 | .428 |
| ratioLSHy | | 1.031 | .486 | -.227 | 1.313 | 1.429 | .161 | .452 |
| **diffLSHy** | | **-1.247** | **-2.424** | **.126** | **2.226** | **2.267** | **.029*** | **.717** |
| ratioRSHy | | .929 | .559 | -.194 | .936 | 1.328 | .192 | .420 |
| **diffRSHy** | | **-1.169** | **-2.329** | **.075** | **2.244** | **2.165** | **.037*** | **.685** |
| ratioLSHx | | .416 | .425 | -.144 | .125 | -.146 | .885 | -.046 |
| diffLSHx | | -2.456 | -3.026 | -.552 | 1.692 | 1.029 | .310 | .325 |
| ratioRSHx | | .600 | .468 | -.105 | .369 | 1.129 | .266 | .357 |
| diffRSHx | | -1.811 | -2.561 | -.228 | 1.724 | 1.557 | .128 | .493 |
| **ratioLSHz** | | **1.393** | **.814** | **.080** | **1.077** | **2.350** | **.024*** | **.743** |
| **diffLSHz** | | **.055** | **-.118** | **.045** | **.302** | **2.724** | **.010*** | **.862** |
| **ratioRSHz** | | **1.352** | **.804** | **.005** | **1.092** | **2.041** | **.048*** | **.646** |
| **diffRSHz** | | **.029** | **-.120** | **.018** | **.279** | **2.303** | **.027*** | **.728** |
| FUB | | 3.059 | 3.202 | -0.691 | 0.405 | -0.528 | 0.601 | -0.167 |
| FUBf | | 1.741 | 2.059 | -0.797 | 0.162 | -1.342 | 0.188 | -0.424 |
| FUBs | | 46.413 | 43.130 | -4.557 | 11.125 | 0.848 | 0.402 | 0.268 |
| FUBt | | 3.226 | 3.513 | -0.732 | 0.159 | -1.300 | 0.201 | -0.411 |
| **g) Variation of Center of Mass** | | | | | | | | |
| VCoMx | | 6.336 | 6.849 | -1.303 | .277 | -1.314 | .197 | -.415 |
| VCoMy | | 6.080 | 5.625 | -.442 | 1.352 | 1.027 | .311 | .325 |
| VCoMz | | 11.111 | 10.884 | -1.611 | 2.065 | .250 | .804 | .079 |
| **h) Velocities of Body Parts** | | | | | | | | |
| **vWSL2** | | **222.366** | **362.129** | **-216.566** | **-62.960** | **-3.684** | **.001**** | **-1.165** |
| **vWSR2** | | **182.898** | **333.890** | **-218.867** | **-83.116** | **-4.503** | **.000***** | **-1.424** |
| **vWSL3** | | **235.064** | **382.036** | **-226.148** | **-67.795** | **-3.758** | **.001**** | **-1.188** |
| **vWSR3** | | **194.879** | **350.584** | **-224.663** | **-86.747** | **-4.571** | **.000***** | **-1.445** |
| **vESL3** | | **126.523** | **184.208** | **-89.630** | **-25.741** | **-3.656** | **.001**** | **-1.156** |
| **vESR3** | | **112.640** | **168.498** | **-82.801** | **-28.917** | **-4.197** | **.000***** | **-1.327** |
| **vKSL3** | | **266.733** | **294.250** | **-53.233** | **-1.800** | **-2.166** | **.037*** | **-.685** |
| **vKSR3** | | **258.404** | **291.409** | **-58.005** | **-8.005** | **-2.673** | **.011*** | **-.845** |
| **i) Relational Velocities of Body Parts** | | | | | | | | |
| **ratiovLKE3** | | **2.244** | **1.731** | **.156** | **.870** | **2.908** | **.006**** | **.919** |
| diffvLKE3 | | 140.210 | 110.042 | -.176 | 60.514 | 2.013 | .051 | .636 |
| **ratiovRKE3** | | **2.482** | **1.846** | **.224** | **1.047** | **3.126** | **.003**** | **.988** |
| diffvRKE3 | | 145.764 | 122.911 | -6.824 | 52.531 | 1.559 | .127 | .493 |

**Note.** Significant differences between the groups are presented in bold. * *p* < 0.05*,* ** *p* < 0.01, ****p* < 0.001. Explanations and definitions of

the movement features are summarized in table 1. LL = Lower Level, UL = Upper Level.

# 2. Auxiliary Analysis

**Table 3. : Auxiliary correlations of the medication load with significant features**

| **Features** | | | | | | | | | | | | | | | |
| --- | --- | --- | --- | --- | --- | --- | --- | --- | --- | --- | --- | --- | --- | --- | --- |
| **Medication Load (OPZ)** | **MV** | **MSTRL** | **MSL** | **alphaHEAD** | **MPower** | **SDPower** | **WSL3** | **WSR3** | **AS3** | **WSL2** | **WSR2** | **AS2** | **ASL1x** | **ASR1x** | **AS1x** |
|  | -0.248 | -0.069 | - 0.069 | -0.034 | 0.059 | 0.305 | 0.103 | 0.293 | 0.221 | 0.104 | 0.296 | 0.224 | 0.088 | 0.245 | 0.188 |
|  | **RatioWSLR** | **ESL3** | **ESR3** | **ES3** | **LBS** | **KSL3** | **KSR3** | **KS3** | **RatioLWEd** | **DiffLWEd** | **RatioRWEd** | **DiffRWEd** | **RatioWEd** | **DiffWEd** | **RatioLAd** |
|  | -0.352 | 0.187 | 0.206 | 0.218 | 0.020 | 0.019 | 0.070 | 0.046 | 0.047 | 0.059 | 0.427 | 0.322 | 0.262 | 0.212 | -0.347 |
|  | **DiffLAd** | **RatioSHd** | **DiffSHd** | **URMLAx** | **URMRAx** | **URMRAy** | **URMLEx** | **URMREx** | **URMLEy** | **URMREy** | **URMLE3** | **URMRE3** | **URMTRy** | **URMLSy** | **URMRSy** |
|  | -0.191 | 0.140 | 0.054 | 0.136 | 0.200 | 0.118 | 0.007 | 0.200 | -0.062 | 0.378 | -0.248 | 0.231 | -0.118 | -0.153 | -0.113 |
|  | **URMLSx** | **URMRSz** | **URMRH2** | **URMRHx** | **URMLHz** | **ratioURMLA** | **diffURMLA** | **diffURMRA** | **diffURMLE** | **ratioURMLS** | **diffURMLS** | **ratioURMRS** | **diffURMRS** | **diffLELAx** | **ratioRERAx** |
|  | 0.054 | -0.307 | 0.275 | -0.058 | 0.180 | 0.244 | 0.133 | 0.188 | 0.025 | 0.235 | 0.190 | -0.056 | -0.080 | -0.078 | 0.303 |
|  | **diffRERAx** | **ratioLELAy** | **diffLELAy** | **ratioRERAy** | **diffRERAy** | **diffLSHy** | **diffRSHy** | **ratioLSHz** | **diffLSHz** | **ratioRSHz** | **diffRSHz** | **vWSL2** | **vWSR2** | **vWSL3** | **vWSR3** |
|  | 0.184 | -0.324 | -0.145 | 0.302 | 0.367 | 0.006 | -0.161 | -0.309 | -0.270 | -0.427 | -0.403 | 0.051 | 0.238 | 0.048 | 0.230 |
|  | **vESL3** | **vESR3** | **vKSL3** | **vKSR3** | **ratiovLKE3** | **ratiovRKE3** |  |  |  |  |  |  |  |  |  |
|  | 0.089 | 0.114 | -0.166 | -0.107 | -0.258 | -0.175 |  |  |  |  |  |  |  |  |  |

**Note.** Correlations follow Pearson and are two-tailed. Significant correlations are presented in bold. * = *p* < 0.05, ** = *p* < 0.01, *** = *p* < 0.001.

**Table 4. Two-sided t-tests comparing patients with high and low medication dose**

| **Feature** | ***M*** | | | **95% CI** | | ***t*_(18)_** | ***p*** | **Cohen’s d** |
| --- | --- | --- | --- | --- | --- | --- | --- | --- |
|  | **OPZ < 17.03**  **(N=10)** | | **OPZ >17.03**  **(N=10)** | **LL** | **UL** |  |  |  |
| **Basic Features** | | | | | | |  |  |
| MV | 1.105 | | 1.064 | -0.099 | 0.182 | 0.622 | 0.541 | 0.278 |
| MSTRL | 1.209 | | 1.220 | -0.144 | 0.123 | -0.169 | 0.868 | -0.076 |
| MSL | 0.605 | | 0.610 | -0.072 | 0.061 | -0.169 | 0.868 | -0.076 |
| Periodicity of Walk | | | | | | | | |
| MPower | | 0.993 | 0.993 | -0.002 | 0.001 | -0.582 | 0.568 | -.0260 |
| SDPower | | 0.001 | 0.001 | -0.000 | 0.000 | -0.888 | 0.386 | -.0397 |
| **Postural Features** | | | | | | | | |
| alphaHEAD | 11.371 | | 14.361 | -9.708 | 3.729 | -0.935 | .362 | -0.418 |
| **Sway of Body Parts** | | | | | | | | |
| WSL3 | | 247.247 | 275.244 | -133.515 | 77.522 | -0.557 | 0.584 | -0.249 |
| WSR3 | | 185.010 | 249.490 | -159.451 | 30.492 | -1.426 | 0.171 | -0.638 |
| AS3 | | 216.129 | 262.367 | -134.517 | 42.041 | -1.100 | 0.286 | -0.492 |
| WSL2 | | 234.336 | 259.837 | -129.974 | 78.971 | -0.513 | 0.614 | -0.229 |
| WSR2 | | 171.213 | 236.433 | -161.216 | 30.776 | -1.427 | 0.171 | -0.638 |
| AS2 | | 202.774 | 248.135 | -134.092 | 43.371 | -1.074 | 0.297 | -0.480 |
| ASL1x | | 229.610 | 250.811 | -124.875 | 82.472 | -0.430 | 0.673 | -0.192 |
| ASR1x | | 166.652 | 225.964 | -158.802 | 40.178 | -1.252 | 0.226 | -0.560 |
| AS1x | | 198.131 | 238.388 | -130.242 | 49.729 | -0.940 | 0.360 | -0.420 |
| ESL3 | | 131.595 | 151.2522 | -54.104 | 14.790 | -1.199 | 0.246 | -0.536 |
| ESR3 | | 114.123 | 137.884 | -58.169 | 10.647 | -1.451 | 0.164 | -0.649 |
| ES3 | | 122.859 | 144.568 | -52.341 | 8.922 | -1.489 | 0.154 | -0.666 |
| LBS | | 40.945 | 42.737 | -9.753 | 6.171 | -0.473 | 0.642 | -0.211 |
| KSL3 | | 296.083 | 302.258 | -42.084 | 29.733 | -0.361 | 0.722 | -0.162 |
| KSR3 | | 282.362 | 297.056 | -50.855 | 21.467 | -0.854 | 0.404 | -0.382 |
| KS3 | | 289.222 | 299.657 | -45.764 | 24.894 | -0.621 | 0.543 | -0.278 |
| **Relational Sway of Body Parts** | | | | | | | | |
| RatioWSLR1 | | 1.512 | 1.3978 | -0.404 | 0.633 | 0.462 | 0.649 | 0.207 |
| RatioLWEd | | 1.802 | 1.7602 | -0.300 | 0.385 | 0.259 | 0.799 | 0.116 |
| **Feature** | | ***M*** | | **95% CI** | | ***t*_(18)_** | ***p*** | **Cohen’s d** |
|  | | **OPZ < 17.03**  **(N=10)** | **OPZ >17.03**  **(N=10)** | **LL** | **UL** |  |  |  |
| DiffLWEd | | 115.652 | 123.992 | -81.032 | 64.353 | -0.241 | 0.812 | -0.108 |
| RatioRWEd | | 1.537 | 1.789 | -0.627 | 0.123 | -1.408 | 0.176 | -0.630 |
| DiffRWEd | | 70.887 | 111.606 | -105.563 | 24.126 | -1.319 | 0.204 | -0.590 |
| RatioWEd | | 1.690 | 1.785 | -0.409 | 0.219 | -0.635 | 0.533 | -0.284 |
| DiffWEd | | 93.270 | 117.799 | -84.896 | 35.834 | -0.854 | 0.405 | -0.382 |
| RatioLAd | | 4.161 | 2.796 | -0.942 | 3.673 | 1.243 | 0.230 | 0.556 |
| DiffLAd | | 416.408 | 372.625 | -71.196 | 158.761 | 0.800 | 0.434 | 0.358 |
| RatioSHd | | 1.609 | 2.146 | -1.452 | 0.377 | -1.236 | 0.233 | -0.553 |
| DiffSHd | | 13.781 | 17.093 | -14.966 | 8.341 | -0.597 | 0.558 | -0.267 |
| **Utilized Range of Motion** | | | | | | | | |
| URMLAx | | 9.168 | 10.338 | -4.589 | 2.248 | -0.719 | 0.481 | -0.322 |
| URMRAx | | 7.559 | 9.723 | -5.498 | 1.168 | -1.364 | 0.189 | -0.610 |
| URMRAy | | 1.461 | 1.597 | -.599 | 0.327 | -0.628 | 0.540 | -0.281 |
| URMLEx | | 19.208 | 19.477 | -8.624 | 8.086 | -0.068 | 0.947 | -0.030 |
| URMREx | | 13.820 | 17.383 | -11.537 | 4.411 | -0.939 | 0.360 | -0.420 |
| URMLEy | | 3.535 | 3.698 | -2.092 | 1.765 | -0.178 | 0.861 | -0.080 |
| URMREy | | 1.865 | 2.443 | -1.682 | 0.525 | -1.101 | 0.285 | -0.492 |
| URMLE3 | | 4.400 | 3.577 | -1.627 | 3.273 | 0.705 | 0.490 | 0.315 |
| URMRE3 | | 3.012 | 4.225 | -3.499 | 1.073 | -1.115 | 0.280 | -0.498 |
| URMTRy | | 1.992 | 1.936 | -0.335 | 0.448 | 0.304 | 0.765 | 0.136 |
| URMLSy | | 1.983 | 1.961 | -0.429 | 0.472 | 0.101 | 0.921 | 0.045 |
| URMRSy | | 1.970 | 1.944 | -0.409 | 0.460 | 0.124 | 0.903 | 0.055 |
| URMLSx | | 1.230 | 1.386 | -0.502 | 0.191 | -0.944 | 0.358 | -0.422 |
| URMRSz | | 0.410 | 0.378 | -0.106 | 0.170 | 0.491 | 0.629 | 0.220 |
| URMRH2 | | 1.087 | 1.307 | -0.760 | 0.320 | -0.875 | 0.397 | -0.391 |
| URMRHx | | 3.589 | 3.423 | -1.136 | 1.466 | 0.266 | 0.793 | 0.119 |
| URMLHz | | 0.332 | 0.370 | -0.162 | 0.086 | -0.639 | 0.531 | -0.286 |
| **Relational Utilized Range of Motion** | | | | | | | | |
| Relation of movement in x (walking direction) and y (sideways) direction | | | | | | | | |
| ratioURMLA | | 4.249 | 4.787 | -2.471 | 1.397 | -0.584 | 0.567 | -0.261 |
| diffURMLA | | 7.021 | 7.790 | -3.839 | 2.300 | -0.526 | 0.605 | -0.235 |
| **Feature** | | ***M*** | | **95% CI** | | ***t*_(18)_** | ***p*** | **Cohen’s d** |
|  | | **OPZ < 17.03**  **(N=10)** | **OPZ >17.03**  **(N=10)** | **LL** | **UL** |  |  |  |
| diffURMRA | | 6.098 | 8.126 | -5.301 | 1.244 | -1.303 | 0.209 | -0.583 |
| diffURMLE | | 15.672 | 15.778 | -7.178 | 6.966 | -0.031 | 0.975 | -0.014 |
| ratioURMLS | | 0.627 | 0.744 | -0.305 | 0.072 | -1.298 | 0.211 | -0.580 |
| diffURMLS | | -0.753 | -0.576 | -0.632 | 0.278 | -0.818 | 0.567 | -0.366 |
| ratioURMRS | | 0.867 | 0.868 | -0.275 | 0.272 | -0.013 | 0.605 | -0.006 |
| diffURMRS | | -0.254 | -0.271 | -0.523 | 0.557 | 0.065 | 0.209 | 0.029 |
| **Relation of movement of different joints** | | | | | | | | |
| diffLELAx | | 10.040 | 9.138 | -4.333 | 6.136 | 0.362 | 0.722 | 0.162 |
| ratioLELAy | | 1.631 | 1.374 | -0.122 | 0.636 | 1.423 | 0.172 | 0.636 |
| diffLELAy | | 1.389 | 1.151 | -0.999 | 1.475 | 0.405 | 0.691 | 0.181 |
| ratioRERAx | | 1.669 | 1.765 | -0.495 | 0.305 | -0.501 | 0.622 | -0.224 |
| diffRERAx | | 6.261 | 7.659 | -6.442 | 3.645 | -0.582 | 0.568 | -0.260 |
| ratioRERAy | | 1.370 | 1.548 | -0.844 | 0.488 | -0.562 | 0.581 | -0.252 |
| diffRERAy | | 0.404 | 0.846 | -1.444 | 0.559 | -0.928 | 0.366 | -0.415 |
| diffLSHy | | -1.383 | -1.112 | -1.686 | 1.144 | -0.402 | 0.692 | -0.180 |
| diffRSHy | | -1.072 | -1.266 | -1.262 | 1.650 | 0.279 | 0.783 | 0.125 |
| ratioLSHz | | 1.556 | 1.230 | -0.577 | 1.229 | 0.759 | 0.458 | 0.339 |
| diffLSHz | | 0.080 | 0.031 | -0.144 | 0.244 | 0.540 | 0.596 | 0.241 |
| ratioRSHz | | 1.628 | 1.075 | -0.422 | 1.527 | 1.192 | 0.249 | 0.533 |
| diffRSHz | | 0.081 | -0.024 | -0.099 | 0.308 | 1.077 | 0.295 | 0.482 |
| **Velocities of Body Parts** | | | | | | | | |
| vWSL2 | | 214.799 | 229.934 | -115.496 | 85.227 | -0.317 | 0.755 | -0.142 |
| vWSR2 | | 158.752 | 207.045 | -138.791 | 42.204 | -1.121 | 0.277 | -0.501 |
| vWSL3 | | 226.625 | 243.503 | -118.622 | 84.866 | -0.349 | 0.731 | -0.156 |
| vWSR3 | | 171.228 | 218.530 | -137.273 | 42.670 | -1.105 | 0.284 | -0.494 |
| vESL3 | | 120.309 | 132.737 | -46.765 | 21.910 | -0.760 | 0.457 | -0.340 |
| vESR3 | | 104.999 | 120.280 | -48.718 | 18.157 | -0.960 | 0.350 | -0.429 |
| vKSL3 | | 270.438 | 263.028 | -29.396 | 44.218 | 0.423 | 0.677 | 0.189 |
| vKSR3 | | 257.932 | 258.875 | -38.918 | 37.034 | -0.052 | 0.959 | -0.023 |
| **Relational Velocities of Body Parts** | | | | | | | | |
| ratiovLKE3 | | 2.401 | 2.088 | -.258 | 0.882 | 1.151 | 0.265 | 0.515 |
| ratiovRKE3 | | 2.657 | 2.307 | -.349 | 1.050 | 1.052 | 0.307 | 0.470 |

**Note.** 17.03 is the median of the medication load (OPZ). Significant differences between the groups are presented in bold. * *p* < 0.05*,* ** *p* < 0.01,

****p* < 0.001. Explanations and definitions of the movement features are summarized in table 1 in the supplementary material.

LL = Lower Level, UL = Upper Level.

**Table 5. : Auxiliary ANCOVA for the control of weight differences**

| **Feature**  **(Dependent Variable)** | **M** | | **Factors**  **(Independent Variable/Covariate)** | **F_(1,37)_** | **p** | **Partial Eta^2^** |
| --- | --- | --- | --- | --- | --- | --- |
|  | **Patient** | **Control** |  |  |  |  |
| **Basic Features** | | | | | | |
| **MV** | **1.084** | **1.209** | **group** | **4.905** | **0.033*** | **0.117** |
|  |  |  | mass | 0.332 | 0.568 | 0.009 |
| **MSTRL** | **1.215** | **1.330** | **group** | **9.142** | **0.005**** | **0.198** |
|  |  |  | mass | 1.857 | 0.181 | 0.048 |
| **MSL** | **0.607** | **0.665** | **group** | **9.142** | **0.005**** | **0.198** |
|  |  |  | mass | 1.857 | 0.181 | 0.048 |
| Periodicity of Walk | | | | | | |
| **MPower** | **0.993** | **0.995** | **group** | **11.181** | **0.002**** | **0.232** |
|  |  |  | mass | 1.806 | 0.187 | 0.047 |
| **SDPower** | **0.0008** | **0.0005** | **group** | **5.969** | **0.019*** | **0.139** |
|  |  |  | mass | 0.737 | 0.713 | 0.020 |
| **Postural Features** | | | | | | |
| **alphaHEAD** | **12.867** | **5.301** | **group** | **12.004** | **0.001**** | **0.245** |
|  |  |  | mass | 2.234 | 0.143 | 0.057 |
| **Sway of Body Parts** | | | | | | |
| **WSL3** | **261.246** | **418.665** | **group** | **12.169** | **0.001**** | **0.247** |
|  |  |  | mass | 0.053 | 0.819 | 0.001 |
| **WSR3** | **217.250** | **384.860** | **group** | **21.851** | **0.000***** | **0.371** |
|  |  |  | mass | 0.799 | 0.377 | 0.021 |
| **AS3** | **239.248** | **401.762** | **group** | **18.941** | **0.000** | **0.339** |
|  |  |  | mass | 0.099 | 0.755 | 0.003 |
| **WSL2** | **247.086** | **396.892** | **group** | **11.563** | **0.002**** | **0.238** |
|  |  |  | mass | 0.076 | 0.785 | 0.002 |
| **WSR2** | **203.823** | **366.547** | **group** | **20.627** | **0.000***** | **0.358** |
|  |  |  | mass | 0.576 | 0.453 | 0.015 |
| **AS2** | **225.55** | **381.719** | **group** | **18.027** | **0.000***** | **0.328** |
|  |  |  | mass | 0.051 | 0.822 | 0.001 |
| **ASL1x** | **240.210** | **392.976** | **group** | **12.379** | **0.001**** | **0.251** |
|  |  |  | mass | 0.064 | 0.802 | 0.002 |
| **ASR1x** | **169.308** | **363.490** | **group** | **21.038** | **0.000***** | **0.362** |
|  |  |  | mass | 0.482 | 0.492 | 0.013 |
| **AS1x** | **218.259** | **378.233** | **group** | **18.872** | **0.000***** | **0.338** |
|  |  |  | mass | 0.045 | 0.833 | 0.001 |
| **ESL3** | **141.424** | **202.882** | **group** | 13.295 | **0.001**** | 0.264 |
|  |  |  | mass | 0.461 | 0.501 | 0.012 |
| **ESR3** | **126.004** | **185.913** | **group** | 20.558 | **0.000***** | 0.357 |
|  |  |  | mass | 2.404 | 0.130 | 0.061 |
| **ES3** | **133.714** | **194.397** | **group** | 18.414 | **0.000***** | 0.332 |
|  |  |  | mass | 1.296 | 0.262 | 0.034 |
| **LBS** | **41.841** | **32.612** | **group** | **5.329** | **0.027*** | **0.126** |
|  |  |  | mass | 4.985 | 0.032 | 0.119 |
| **HS3** | **61.308** | **67.400** | **group** | **5.807** | **0.021*** | **0.136** |
|  |  |  | mass | 3.261 | 0.079 | 0.081 |
| **KSL3** | **299.171** | **323.829** | **group** | **5.514** | **0.024*** | **0.130** |
|  |  |  | mass | 1.248 | 0.271 | 0.033 |
| **KSR3** | **289.709** | **321.029** | **group** | **9.174** | **0.004**** | **0.199** |
|  |  |  | mass | 2.082 | 0.157 | 0.053 |
| **KS3** | **294.440** | **322.429** | **group** | **7.599** | **0.009**** | **0.170** |
|  |  |  | mass | 1.723 | 0.197 | 0.044 |
| **Relational Sway of Body Parts** | | | | | | |
| **RatioWSLR1** | **1,753** | **1,255** | **group** | **4.327** | **0.043*** | **0.106** |
|  |  |  | mass | 0.161 | 0.691 | 0.004 |
| **RatioWEd** | **1.738** | **2.062** | **group** | **7.011** | **0.012*** | **0.159** |
|  |  |  | mass | 2.030 | 0.163 | 0.052 |
| **DiffWEd** | **105.534** | **207.365** | **group** | **16.784** | **0.000***** | **0.312** |
|  |  |  | mass | 0.053 | 0.819 | 0.001 |
| **RatioLAd** | **3.479** | **1.929** | **group** | **5.519** | **0.024*** | **0.130** |
|  |  |  | mass | 0.216 | 0.645 | 0.006 |
| **DiffLAd** | **394.516** | **282.596** | **group** | **6.849** | **0.013*** | **0.156** |
|  |  |  | mass | 0.958 | 0.334 | 0.025 |
| RatioSHd | 1.878 | 1.172 | group | 3.698 | 0.062 | 0.091 |
|  |  |  | mass | 4.633 | 0.038* | 0.111 |
| **DiffSHd** | **15.437** | **1.007** | **group** | **5.408** | **0.026*** | **0.128** |
|  |  |  | mass | 4.681 | 0.037* | 0.112 |
| **Utilized Range of Motion** | | | | | | |
| **URMLAx** | **9,753** | **15,027** | **group** | **10,735** | **,002**** | **,225** |
|  |  |  | mass | ,012 | ,914 | ,000 |
| **URMRAx** | **8,641** | **13,647** | **group** | **16,213** | **,000**** | **,305** |
|  |  |  | mass | ,656 | ,423 | ,017 |
| URMLAy | 2,347 | 2,561 | group | ,148 | ,702 | ,004 |
|  |  |  | mass | 1,014 | ,320 | ,027 |
| **URMRAy** | **1,529** | **2,043** | **group** | **6,280** | **,017**** | **,145** |
|  |  |  | mass | ,182 | ,672 | ,005 |
| **URMLEx** | **19,342** | **30,572** | **group** | **9,944** | **,003**** | **,212** |
|  |  |  | mass | 2,053 | ,160 | ,053 |
| **URMREx** | **15,601** | **28,653** | **group** | **18,111** | **,000***** | **,329** |
|  |  |  | mass | ,072 | ,790 | ,002 |
| **URMLEy** | **3,617** | **6,254** | **group** | **6,486** | **,015*** | **,149** |
|  |  |  | mass | 2,527 | ,120 | ,064 |
| **URMREy** | **2,154** | **5,072** | **group** | **18,780** | **,000***** | **,337** |
|  |  |  | mass | 1,411 | ,243 | ,037 |
| **URMLE3** | **3,988** | **6,485** | **group** | **4,800** | **,035*** | **,115** |
|  |  |  | mass | 1,610 | ,212 | ,042 |
| **URMRE3** | **3,618** | **6,956** | **group** | **10,692** | **,002**** | **,224** |
|  |  |  | mass | ,004 | ,950 | ,000 |
| **URMTRy** | **1,964** | **1,559** | **group** | **5,996** | **,019*** | **,139** |
|  |  |  | mass | 1,162 | ,288 | ,030 |
| URMLSy | 1,972 | 1,656 | group | 2,946 | ,094 | ,074 |
|  |  |  | mass | ,313 | ,579 | ,008 |
| URMRSy | 1,958 | 1,590 | group | 3,795 | ,059 | ,093 |
|  |  |  | mass | ,715 | ,403 | ,019 |
| **URMLSx** | **1,308** | **1,757** | **group** | **17,267** | **,000***** | **,318** |
|  |  |  | mass | ,697 | ,409 | ,018 |
| URMRSx | 1,695 | 1,926 | group | ,607 | ,441 | ,016 |
|  |  |  | mass | 1,624 | ,210 | ,042 |
| URMLSz | ,407 | ,320 | group | 2,590 | ,116 | ,065 |
|  |  |  | mass | ,079 | ,780 | ,002 |
| URMRSz | ,394 | ,298 | group | 2,787 | ,103 | ,070 |
|  |  |  | mass | ,635 | ,430 | ,017 |
| URMLH2 | 1,298 | 1,503 | group | 1,130 | ,295 | ,030 |
|  |  |  | mass | ,002 | ,967 | ,000 |
| **URMRH2** | **1,197** | **1,584** | **group** | **4,555** | **,040*** | **,110** |
|  |  |  | mass | ,115 | ,736 | ,003 |
| URMLHy | 3,219 | 4,079 | group | 1,659 | ,206 | ,043 |
|  |  |  | mass | 3,388 | ,074 | ,084 |
| URMRHy | 3,127 | 3,919 | group | 1,734 | ,196 | ,045 |
|  |  |  | mass | 1,417 | ,241 | ,037 |
| URMLHx | 3,764 | 4,783 | group | 1,745 | ,195 | ,045 |
|  |  |  | mass | 1,765 | ,192 | ,046 |
| URMRHx | 3,506 | 4,487 | group | 2,681 | ,110 | ,068 |
|  |  |  | mass | 2,536 | ,120 | ,064 |
| **URMLHz** | **,351** | **,437** | **group** | **4,884** | **,033*** | **,117** |
|  |  |  | mass | ,656 | ,423 | ,017 |
| URMRHz | ,365 | ,418 | group | 3,005 | ,091 | ,075 |
|  |  |  | mass | 2,893 | ,097 | ,073 |
| **Relational Utilized Range of Motion** | | | | | | |
| Relation of movement in x (walking direction) and y (sideways) direction | | | | | | |
| **ratioURMLA** | **4,518** | **6,212** | **group** | **6,614** | **,014*** | **,152** |
|  |  |  | mass | ,604 | ,442 | ,016 |
| **diffURMLA** | **7,406** | **12,466** | **group** | **12,151** | **,001**** | **,247** |
|  |  |  | mass | ,007 | ,932 | ,000 |
| ratioURMRA | 6,068 | 7,364 | group | 2,108 | ,155 | ,054 |
|  |  |  | mass | ,450 | ,506 | ,012 |
| **diffURMRA** | **7,112** | **11,604** | **group** | **13,531** | **,001**** | **,268** |
|  |  |  | mass | ,768 | ,387 | ,020 |
| ratioURMLE | 5,993 | 5,488 | group | ,244 | ,624 | ,007 |
|  |  |  | mass | ,110 | ,742 | ,003 |
| **diffURMLE** | **15,725** | **24,318** | **group** | **9,223** | **,004**** | **,200** |
|  |  |  | mass | 1,515 | ,226 | ,039 |
| ratioURMRE | 7,807 | 6,104 | group | 2,161 | ,150 | ,055 |
|  |  |  | mass | ,248 | ,621 | ,007 |
| **diffURMRE** | **6,487** | **8,575** | **group** | **5,881** | **,020*** | **,137** |
|  |  |  | mass | 2,675 | ,110 | ,067 |
| **ratioURMLS** | **,685** | **1,152** | **group** | **17,238** | **,000***** | **,318** |
|  |  |  | mass | ,660 | ,422 | ,018 |
| **diffURMLS** | **-,664** | **,101** | **group** | **19,846** | **,000***** | **,349** |
|  |  |  | mass | ,000 | ,983 | ,000 |
| **ratioURMRS** | **,867** | **1,330** | **group** | **7,298** | **,010*** | **,165** |
|  |  |  | mass | 6,979 | ,012* | ,159 |
| **diffURMRS** | **-,262** | **,337** | **group** | **6,561** | **,015*** | **,151** |
|  |  |  | mass | 4,369 | ,044* | ,106 |
| Relation of movement of different joints | | | | | | |
| ratioLELAx | 1,934 | 2,103 | **group** | ,164 | ,688 | ,004 |
|  |  |  | mass | 8,915 | ,005 | ,194 |
| **diffLELAx** | **9,589** | **15,545** | **group** | **7,070** | **,012*** | **,160** |
|  |  |  | mass | 6,212 | ,017 | ,144 |
| **ratioLELAy** | **1,502** | **2,468** | **group** | **10,769** | **,002**** | **,225** |
|  |  |  | mass | 2,697 | ,109 | ,068 |
| **diffLELAy** | **1,270** | **3,693** | **group** | **9,723** | **,004**** | **,208** |
|  |  |  | mass | 2,571 | ,117 | ,065 |
| **ratioRERAx** | **1,717** | **2,125** | **group** | **6,265** | **,017*** | **,145** |
|  |  |  | mass | 2,384 | ,131 | ,061 |
| **diffRERAx** | **6,960** | **15,006** | **group** | **15,285** | **,000***** | **,292** |
|  |  |  | mass | ,983 | ,328 | ,026 |
| **ratioRERAy** | **1,459** | **2,625** | **group** | **9,891** | **,003**** | **,211** |
|  |  |  | mass | ,931 | ,341 | ,025 |
| **diffRERAy** | **,625** | **3,029** | **group** | **15,287** | **,000***** | **,292** |
|  |  |  | mass | 1,350 | ,253 | ,035 |
| ratioLSHy | 1,031 | ,486 | group | 1,448 | ,236 | ,038 |
|  |  |  | mass | ,155 | ,696 | ,004 |
| diffLSHy | -1,247 | -2,424 | group | 2,636 | ,113 | ,067 |
|  |  |  | mass | 3,002 | ,092 | ,075 |
| ratioRSHy | ,929 | ,559 | group | 1,254 | ,270 | ,033 |
|  |  |  | mass | ,131 | ,720 | ,004 |
| diffRSHy | -1,169 | -2,329 | group | 2,730 | ,107 | ,069 |
|  |  |  | mass | 1,502 | ,228 | ,039 |
| **ratioLSHz** | **1,393** | **,814** | **group** | **5,536** | **,024*** | **,130** |
|  |  |  | mass | ,227 | ,637 | ,006 |
| **diffLSHz** | **,055** | **-,118** | **group** | **7,002** | **,012*** | **,159** |
|  |  |  | mass | ,101 | ,752 | ,003 |
| **ratioRSHz** | **1,352** | **,804** | **group** | **4,639** | **,038*** | **,111** |
|  |  |  | mass | ,524 | ,473 | ,014 |
| **diffRSHz** | **,029** | **-,120** | **group** | **5,457** | **,025*** | **,129** |
|  |  |  | mass | ,305 | ,584 | ,008 |
| **Velocity of Body Parts** | | | | | | |
| **vWSL2** | **222,366** | **362,129** | **group** | **10,163** | **,003**** | **,215** |
|  |  |  | mass | ,695 | ,410 | ,018 |
| **vWSR2** | **182,898** | **333,890** | **group** | **18,156** | **,000***** | **,329** |
|  |  |  | mass | ,038 | ,846 | ,001 |
| **vWSL3** | **235,064** | **382,036** | **group** | **10,668** | **,002**** | **,224** |
|  |  |  | mass | ,638 | ,429 | ,017 |
| **vWSR3** | **194,879** | **350,584** | **group** | **19,019** | **,000***** | **,340** |
|  |  |  | mass | ,089 | ,767 | ,002 |
| **vESL3** | **126,523** | **184,208** | **group** | **11,454** | **,002**** | **,236** |
|  |  |  | mass | ,004 | ,951 | ,000 |
| **vESR3** | **112,640** | **168,498** | **group** | **17,520** | **,000***** | **,321** |
|  |  |  | mass | ,560 | ,459 | ,015 |
| vKSL3 | 266,733 | 294,250 | group | 3,299 | ,077 | ,082 |
|  |  |  | mass | ,413 | ,524 | ,011 |
| **vKSR3** | **258,404** | **291,409** | **group** | **5,678** | **,022*** | **,133** |
|  |  |  | mass | ,111 | ,741 | ,003 |
| **Relational Velocities of Body Parts** | | | | | | |
| **ratiovLKE3** | **2,244** | **1,731** | **group** | **7,448** | **,010*** | **,168** |
|  |  |  | mass | ,004 | ,950 | ,000 |
| **diffvLKE3** | **140,210** | **110,042** | **group** | **4,153** | **,049*** | **,101** |
|  |  |  | mass | ,228 | ,636 | ,006 |
| **ratiovRKE3** | **2,482** | **1,846** | **group** | **9,505** | **,004**** | **,204** |
|  |  |  | mass | ,242 | ,626 | ,006 |
| diffvRKE3 | 145,764 | 122,911 | group | 3,202 | ,082 | ,080 |
|  |  |  | mass | ,934 | ,340 | ,025 |

**Note.** Significant differences between the groups are presented in bold. * *p* < 0.05*,* ** *p* < 0.01, ****p* < 0.001. Explanations and definitions of the

movement features are summarized in table 1.

3. Matching Procedure

We chose logistic regression for the estimation of propensity scores and created a matched sample using the one-to-one approach (Caliendo and Kopeinig, 2008; Harris and Horst, 2016; Ejdemyr, 2020). Except for the variable gender (exact matching), we chose nearest-neighbor matching. Because there are no inferences made in relation to a population, Harris and Horst (2016) consider t-tests an inappropriate tool to evaluate covariate balance after matching. Hence, as suggested by Stuart (2010) we compared group means after matching and calculated variance ratios. According to Rubin and Ho et al. (Rubin, 2001; Ho et al., 2007) group means should not differ more than one-fourth of a standard deviation on any of the covariates and variance ratios should be close to one. In our case, group means of mass and BMI differed more than one-fourth a standard deviation but all variance ratios were close to one (see tables below). Hence, after balance comparison, we based the main analysis on the resulting matched and reduced sample and controlled for the variable mass within the data-based exploration of movement patterns and within the auxiliary analysis.

| **Table 6. Comparison of Means before Matching** | | | | | | |
| --- | --- | --- | --- | --- | --- | --- |
| **Group** | **Participants** | **BMI** | **Age** | **Height** | **Mass** | **Gender** |
| Patient | 20 | 29.01 | 39.00 | 177.04 | 91.42 | 0.30 |
| Control | 26 | 24.52 | 36.96 | 178.48 | 78.40 | 0.35 |
| **Note**. Means of patient and control group previous to matching. | | | | | | |
|  | | | | | | |

| **Table 7. T-Test Before Matching** | | | | | | | |
| --- | --- | --- | --- | --- | --- | --- | --- |
|  | **Means** | |  | | | **95% CI** | |
| **Covariate** | **Patient** | **Control** | **t** | **p** | **df** | **LL** | **UL** |
| Gender | 0.30 | 0.35 | -0.33 | 0.75 | 41.64 | -0.33 | 0.24 |
| Age | 39.00 | 36.96 | 0.61 | 0.55 | 38.49 | -4.77 | 8.85 |
| Height | 177.04 | 178.48 | -0.51 | 0.61 | 39.17 | -7.18 | 4.29 |
| Mass | 91.42 | 78.40 | 2.83 | 0.01 | 38.86 | 3.72 | 22.33 |
| BMI | 29.01 | 24.52 | 4.34 | 0.00 | 42.41 | 2.40 | 6.57 |
| **Note**. Two-sided t-test of all covariates before matching. | | | | | | | |

| **Table 8. Estimated Propensity Scores** | | |
| --- | --- | --- |
| **Propensity Score** | **Group** | **ID** |
| 0.62 | 1 | AB04 |
| 0.85 | 1 | AK03 |
| 0.53 | 1 | BB05 |
| 0.96 | 1 | CF12 |
| 0.47 | 1 | CL08 |
| 0.84 | 1 | CM10 |
| 0.34 | 1 | CS01 |
| 0.56 | 1 | DF01 |
| 0.72 | 1 | EH04 |
| 0.58 | 1 | HA10 |
| 0.34 | 1 | IH09 |
| 0.56 | 1 | IH12 |
| 0.92 | 1 | KB04 |
| 0.38 | 1 | KH04 |
| 0.40 | 1 | NH07a |
| 0.80 | 1 | PT08 |
| 0.27 | 1 | RH04 |
| 0.82 | 1 | RM05 |
| 0.70 | 1 | SF03a |
| 0.71 | 1 | SF03b |
| 0.08 | 0 | AG04 |
| 0.14 | 0 | AH01 |
| 0.06 | 0 | BB01 |
| 0.05 | 0 | BB09 |
| 0.95 | 0 | BK06 |
| 0.19 | 0 | CA09 |
| 0.27 | 0 | DD12 |
| 0.10 | 0 | EB08 |
| 0.42 | 0 | EP07 |
| 0.62 | 0 | GH09 |
| 0.10 | 0 | GS05 |
| 0.08 | 0 | HH07 |
| 0.15 | 0 | HM05 |
| 0.66 | 0 | IH04 |
| 0.12 | 0 | JH11 |
| 0.65 | 0 | KK10 |
| 0.09 | 0 | LB12 |
| 0.28 | 0 | ML12 |
| 0.39 | 0 | NH07b |
| 0.06 | 0 | PH02 |
| 0.69 | 0 | PR01 |
| 0.43 | 0 | RB03 |
| 0.08 | 0 | SH01 |
| 0.21 | 0 | SR10 |
| 0.13 | 0 | SV12 |
| 0.61 | 0 | SW02 |

**Note.** Propensity scores were estimated

using logistic regression. 1 = patient

group, 0 = control group. None of the

covariates (gender, age, mass, height,

BMI) served as a significant predictor

of the assigned group of the participants.

| **Table 9. Matched Pairs** | | | |
| --- | --- | --- | --- |
| **Patients** | **Controls** | **Patients** | **Controls** |
| 1 | 38 | AB04 | ML12 |
| 2 | 36 | AK03 | KK10 |
| 3 | 22 | BB05 | AH01 |
| 4 | 25 | CF12 | BK06 |
| 5 | 26 | CL08 | CA09 |
| 6 | 34 | CM10 | IH04 |
| 7 | 45 | CS01 | SV12 |
| 8 | 44 | DF01 | SR10 |
| 9 | 42 | EH04 | RB03 |
| 10 | 27 | HA10 | DD12 |
| 11 | 37 | IH09 | LB12 |
| 12 | 33 | IH12 | HM05 |
| 13 | 41 | KB04 | PR01 |
| 14 | 31 | KH04 | GS05 |
| 15 | 28 | NH07a | EB08 |
| 16 | 46 | PT08 | SW02 |
| 17 | 32 | RH04 | HH07 |
| 18 | 30 | RM05 | GH09 |
| 19 | 39 | SF03a | NH07b |
| 20 | 29 | SF03b | EP07 |

**Note**. The table shows matched Pairs

of patient and control Group. The

first two columns are the serial numbers

of the individuals, the second two their

IDs.


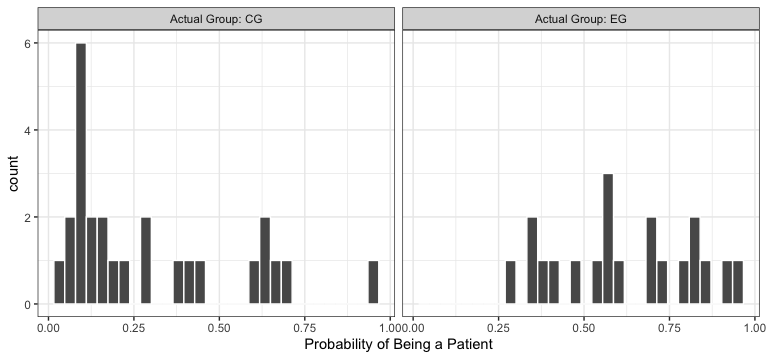


**Figure 1*.*** *Probability of being a patient in both groups calculated according to the five covariates matching was done with. Probability was calculated before matching. In the control group there is many individuals with a very low probability of being a patient.*


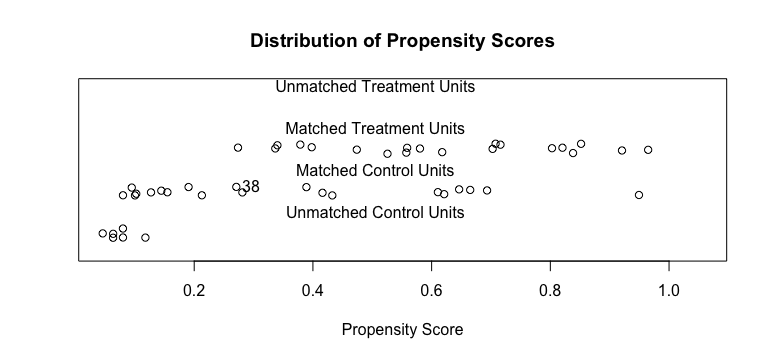


**Figure 2.** *Jitterplot comparing patient and control group in their propensity scores after matching.*

*The jitterplot also displays the excluded (unmatched) units. Controls with very low probability of having*

*the diagnosis Schizophrenia (according to the covariates) were excluded. A total of 20 patient units was*

*matched to 20 control units; 8 control units were discarded.*

Summary of balance for all data:

Means Treated Means Control SD Control Mean Diff eQQ Med eQQ Mean eQQ Max

distance 0.6183 0.2936 0.2560 0.3247 0.3530 0.3328 0.4898

sex 0.3000 0.3462 0.4852 -0.0462 0.0000 0.0500 1.0000

age 39.0000 36.9615 10.5848 2.0385 2.5000 2.8500 7.0000

height 177.0400 178.4846 9.0824 -1.4446 1.9500 2.3700 9.0000

mass 91.4250 78.3962 14.6102 13.0288 15.5500 13.9200 21.9000

BMI 29.0071 24.5198 3.6205 4.4873 4.5772 4.5933 6.0274

Summary of balance for matched data:

Means Treated Means Control SD Control Mean Diff eQQ Med eQQ Mean eQQ Max

distance 0.6183 0.3592 0.2579 0.2592 0.2736 0.2592 0.3708

sex 0.3000 0.3000 0.4702 0.0000 0.0000 0.0000 0.0000

age 39.0000 38.2000 11.1100 0.8000 1.0000 1.6000 6.0000

height 177.0400 177.7500 9.2176 -0.7100 1.9500 2.3400 9.0000

mass 91.4250 80.7300 15.2570 10.6950 9.5000 10.6950 20.5000

BMI 29.0071 25.4350 3.6198 3.5721 3.4757 3.5721 4.9611

Percent Balance Improvement:

Mean Diff. eQQ Med eQQ Mean eQQ Max

distance 20.1896 22.5058 22.1174 24.3026

sex 100.0000 0.0000 1 00.0000 100.0000

age 60.7547 60.0000 43.8596 14.2857

height 50.8520 0.0000 1.2658 0.0000

mass 17.9129 38.9068 23.1681 6.3927

BMI 20.3954 24.0643 22.2330 17.6912

Sample sizes:

Control Treated

All 26 20

Matched 20 20

Unmatched 6 0

Discarded 0 0

| **Table 10. Covariate Balance after Matching** | | | | | | | | | | |
| --- | --- | --- | --- | --- | --- | --- | --- | --- | --- | --- |
| **Mean** | | | | | | **SD** | | | | |
| **Group** | **Gender** | **Age** | **Height** | **Mass** | **BMI** | **Gender** | **Age** | **Height** | **Mass** | **BMI** |
| Control | 0.30 | 38.20 | 177.75 | 80.73 | 25.44 | 0.47 | 11.11 | 9.22 | 15.26 | 3.62 |
| Patient | 0.30 | 39.00 | 177.04 | 91.42 | 29.01 | 0.47 | 11.84 | 9.87 | 16.09 | 3.36 |
| Note. Covariate Balance after Matching. Displayed are Means and Standard Deviations. | | | | | | | | | | |

| **Table 11. Covariate Balance after Matching** | | | | |
| --- | --- | --- | --- | --- |
| (M/SD) | **Control** | **Patient** | **SMD** | **Variance Ratio** |
| **n** | 20 | 20 |  |  |
| **Gender** | 0.30 (0.47) | 0.30 (0.47) | <0.001 | 1 |
| **Age** | 38.20 (11.11) | 39.00 (11.84) | 0.070 | 0.94 |
| **Height** | 177.75 (9.22) | 177.04 (9.87) | 0.074 | 0.93 |
| **Mass** | 80.73 (15.26) | 91.42 (16.09) | 0.682 | 0.95 |
| **BMI** | 25.44 (3.62) | 29.01 (3.36) | 1.023 | 1.08 |
| **Note**. Covariate Balance after Matching. Displayed are Means, Standard Deviations and the Standardized Mean Difference. A standardized Mean difference below 0.1 is considered balanced. Variance ratios close to one are considered balanced(Harris and Horst, 2016). | | | | |

| **Table 12. T-Test After Matching** | | | | | | | |
| --- | --- | --- | --- | --- | --- | --- | --- |
|  | **Means** | |  | | | **95% CI** | |
| **Covariate** | **Patient** | **Control** | **t** | **p** | **df** | **LL** | **UL** |
| Gender | 0.30 | 0.30 | 0.00 | 1.00 | 38.00 | -0.30 | 0.30 |
| Age | 39.00 | 38.20 | 0.22 | 0.83 | 37.85 | -6.55 | 8.15 |
| Height | 177.04 | 177.75 | -0.24 | 0.82 | 37.82 | -6.83 | 5.41 |
| Mass | 91.42 | 80.73 | 2.16 | 0.04 | 37.89 | 0.66 | 20.73 |
| BMI | 29.01 | 25.44 | 3.24 | 0.00 | 37.79 | 1.34 | 5.81 |
| **Note**. Two-sided t-test of all covariates after matching. | | | | | | | |


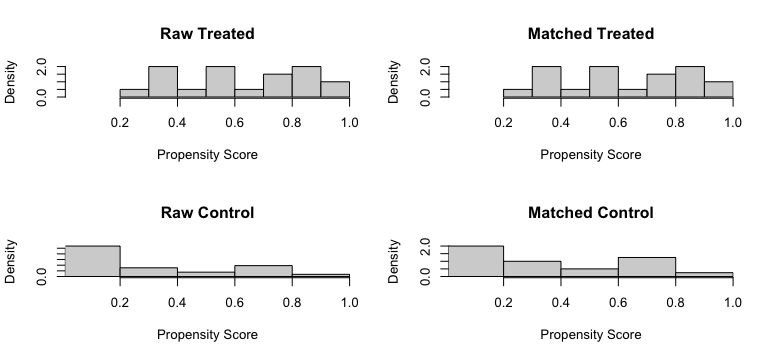


**Figure 3.** *Histograms comparing distributions of propensity scores of patient and control group before and after matching.*


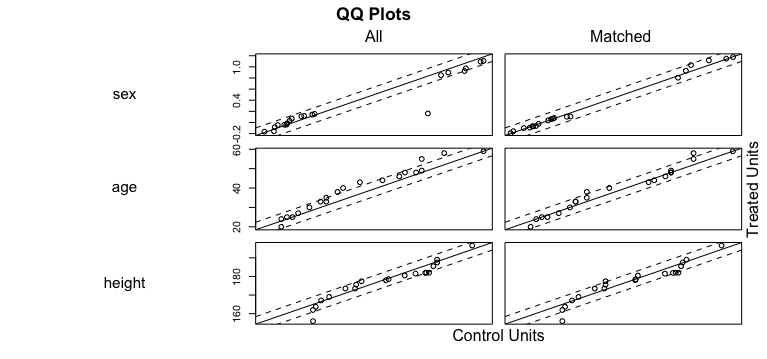


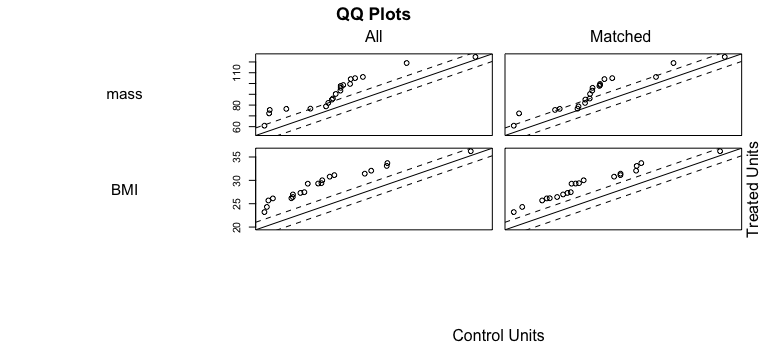

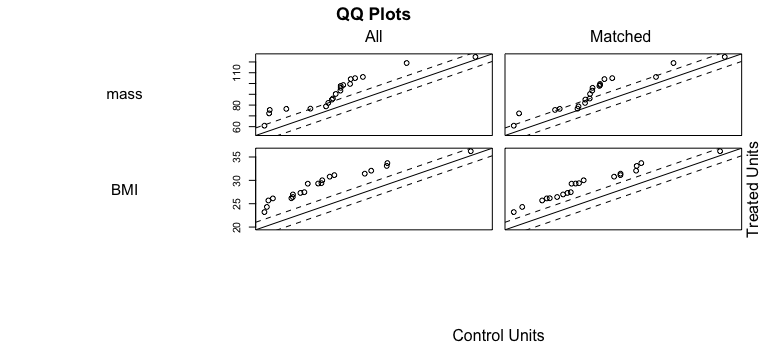


**Figure 4**. *Probability distribution devided into quantiles. In all variables but mass and BMI the majority of the points remain near the center line for the matched QQ plots (right column). This indicates that patients and their matches had similar scores on the respective covariates.*

References

Caliendo, M. and S. Kopeinig (2008). "SOME PRACTICAL GUIDANCE FOR THE IMPLEMENTATION OF PROPENSITY SCORE MATCHING." Journal of Economic Surveys **22**(1): 31-72.

Ejdemyr, S. (2020). "R Tutorial 8: Propensity Score Matching." Retrieved 15.10.2020, from <https://sejdemyr.github.io/r-tutorials/statistics/tutorial8.html>.

Harris, H. and S. J. Horst (2016). "A brief guide to decisions at each step of the propensity score matching process." Practical Assessment, Research, and Evaluation **21**(1): 4.

Ho, D. E., K. Imai, G. King and E. A. Stuart (2007). "Matching as nonparametric preprocessing for reducing model dependence in parametric causal inference." Political analysis **15**(3): 199-236.

Rubin, D. B. (2001). "Using Propensity Scores to Help Design Observational Studies: Application to the Tobacco Litigation." Health Services & Outcomes Research Methodology **2**: 169–188.

Stuart, E. A. (2010). "Stuart, E. A. (2010). Matching methods for causal inference: A review and a look forward." Statistical science: a review journal of the Institute of Mathematical Statistics **25**(1).
